# Novel Photo- and Thermo-Responsive Nanocomposite Hydrogels Based on Functionalized rGO and Modified SIS/Chitosan Polymers for Localized Treatment of Malignant Cutaneous Melanoma

**DOI:** 10.3389/fbioe.2022.947616

**Published:** 2022-07-06

**Authors:** Daniela N. Céspedes-Valenzuela, Santiago Sánchez-Rentería, Javier Cifuentes, Saul C. Gómez, Julian A. Serna, Laura Rueda-Gensini, Carlos Ostos, Carolina Muñoz-Camargo, Juan C. Cruz

**Affiliations:** ^1^ Grupo de Investigación en Nanobiomateriales, Ingeniería Celular y Bioimpresión (GINIB), Department of Biomedical Engineering, Universidad de Los Andes, Bogotá, Colombia; ^2^ Grupo CATALAD, Instituto de Química, Universidad de Antioquia, Medellín, Colombia

**Keywords:** melanoma, photothermal therapy, methacrylation, reduced graphene oxide, COMSOL multiphysics

## Abstract

Melanoma is an aggressive type of skin cancer that accounts for over 75% of skin cancer deaths despite comprising less than 5% of all skin cancers. Despite promising improvements in surgical approaches for melanoma resection, the survival of undetectable microtumor residues has remained a concern. As a result, hyperthermia- and drug-based therapies have grown as attractive techniques to target and treat cancer. In this work, we aim to develop a stimuli-responsive hydrogel based on chitosan methacrylate (ChiMA), porcine small intestine submucosa methacrylate (SISMA), and doxorubicin-functionalized reduced graphene oxide (rGO-DOX) that eliminates microtumor residues from surgically resected melanoma through the coupled effect of NIR light-induced photothermal therapy and heat-induced doxorubicin release. Furthermore, we developed an *in silico* model to optimize heat and mass transport and evaluate the proposed chemo/photothermal therapy *in vitro* over melanoma cell cultures.

## Introduction

Melanoma is a type of skin cancer that arises from the malignant transformation of melanocytes, cells derived from the neural crest stem cells (NCSCs) that produce melanin in the skin. From the several existing types of melanomas, cutaneous melanoma is recognized as the most prevalent (Rebecca et al., 2020). The severity of melanoma resides in it accounting for over 75% of the total skin cancer deaths despite comprising less than 5% of all skin cancers (Cummins et al., 2006; Shenenberger, 2012). The NCSC origin of melanocytes has been thought to be responsible for the ability of melanoma to both migrate and thrive in major organs, such as the brain and the lungs (Rebecca et al., 2020). Moreover, its characteristic high proliferation rate and early metastasis to the lymphatic and blood tracts cause over 60% of melanoma patients to fail to respond adequately to current treatments such as radiotherapy and chemotherapy (Joyce and Skitzki, 2020; Sandru et al., 2020). Despite recent improvements in the diagnosis, staging, classification, and treatment of cutaneous melanoma, the development of novel approaches to target the persisting melanoma cells remains challenging (Coit et al., 2019; Yang et al., 2019).

Surgical approaches for skin cancer are generally based on only resecting the visible tumor in an attempt to preserve the largest possible area of healthy tissue, as removing large portions of the skin may lead to compromising critical homeostatic functions (Thomas et al., 2003; Blanpain and Fuchs, 2009). However, this approach might result in an increased risk for cancer recurrence from the settlement and proliferation of undetected malignant cells (Hocevar et al., 2014; Nosrati et al., 2017). To overcome this difficulty, Dr. Frederick Mohs proposed an alternative procedure in which thin layers were circumferentially excised around the clinical margins of the tumor until no cancer cells were identified in their histology micrographs (Joyce and Skitzki, 2020; Prickett and Ramsay, 2022). Mohs micrographic chemosurgery has considerably improved the outcome of melanoma patients over the last decades, but the everlasting concern for the survival of microtumor residues has led to the development of complementary novel techniques for cancer treatment, such as hyperthermia- and drug-based therapies (Coit et al., 2019; Joyce and Skitzki, 2020).

Hyperthermia refers to the local increment of temperature to induce protein denaturation, membrane destabilization, and cell necrosis and apoptosis (Zhu et al., 2012). Reports have shown that cancer cells are highly sensitive to hyperthermia, making it an attractive approach for cancer ablation if properly focused over malignant tissue (DeNardo and DeNardo, 2008; Sarkar et al., 2015). In photothermal therapy, which is based on the conversion of light into heat by a photothermal transduction agent (PTA), it is possible to focus the collimated beam of a laser in the near-infrared (NIR) spectrum on a specific population of cells, thus achieving the localized heating of a tumor without significantly damaging the adjacent healthy tissue (Shao et al., 2018; Vines et al., 2019; Zhang et al., 2019). This type of application is particularly useful for skin cancer since light stimuli will not be attenuated by tissue penetration (Fernandez-Villamarin et al., 2019). Among the most common PTA, graphene oxide (GO) and reduced graphene oxide (rGO) have stood out not only by their promising photothermal activity and thermal conductivity but also by their potential to help mimic the mechanical properties of tissue microenvironments and promote tissue regeneration when embedded in three-dimensional constructs for regenerative purposes (Kim et al., 2013; Hu et al., 2018; Dash et al., 2021). Moreover, the high abundance of oxygen-rich functional groups on their surface makes them suitable supports for protein adsorption and drug conjugation and delivery (Campbell et al., 2019; Zhang et al., 2019; Karki et al., 2020; Raslan et al., 2020; Patarroyo et al., 2021).

Drug-based strategies for cancer treatment focus on the delivery and controlled release of specific pharmaceutical compounds that attack cancer cells through different biomolecular mechanisms (Kankala et al., 2019). Doxorubicin (DOX), for instance, generates free radicals that cause oxidative damage to biomolecules and suppress replication and transcription by intercalating within DNA strands and by blocking the topoisomerase II (Taymaz-Nikerel et al., 2018; Johnson-Arbor and Duber, 2021). In recent years, hydrogels have been extensively studied as drug delivery platforms, as they can be tailored to achieve specific release profiles. Moreover, the development of novel “smart” hydrogels has expanded their scope, as they have enabled delivery systems that respond to physical and chemical stimuli, both of natural and artificial origin (Dadsetan et al., 2010; Li and Mooney, 2016; Askari et al., 2020). Among these, light-responsive hydrogels have drawn particular attention, as fine rheological tuning through photo-crosslinking has been employed for bioprinting and *in situ* deposition applications (Serna et al., 2019; Kim et al., 2020). Furthermore, when coupled with other stimuli-responsive components, these multifunctional materials provide an opportunity for implementing potent multimodal therapies (Chen et al., 2019; Huang et al., 2020). For instance, several authors have reported the use of light-induced hyperthermia to promote drug release and diffusion, an approach also commonly known as chemo/photothermal therapy (Shao et al., 2018; Hou et al., 2019; Lima-Sousa et al., 2020).

**FIGURE 1:**
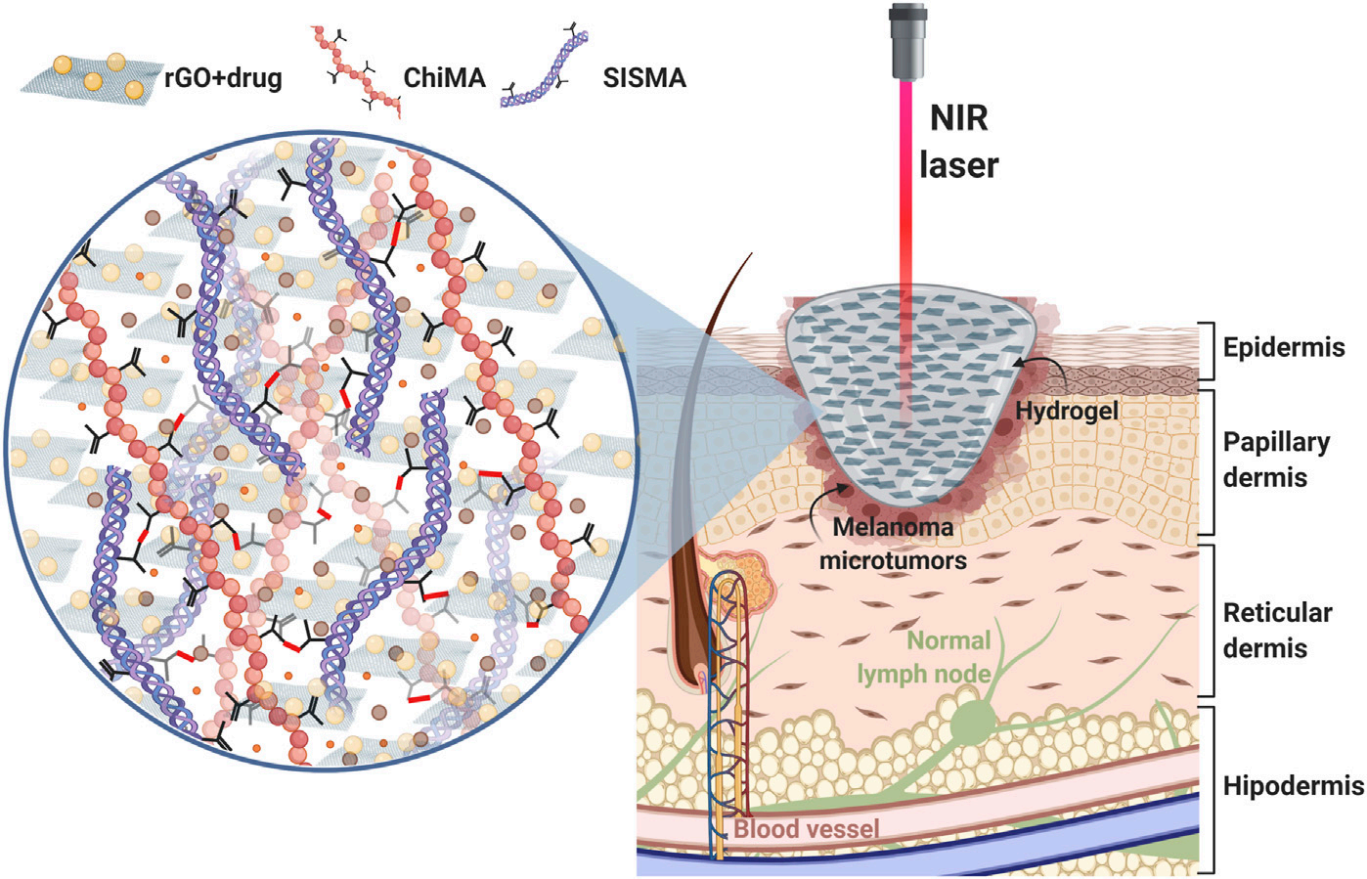
Proposed approach: a SISMA/ChiMA/rGO composite hydrogel employed as a platform for chemo/photothermal therapy to target microtumor residues that remain after the surgical resection of cutaneous melanoma. When stimulated with a NIR laser, the rGO in the hydrogel generates local hyperthermia by converting light into heat. Subsequently, DOX is released from the hydrogel, as the increasing temperature breaks the azo bonds that link the anticancer drug to the GO vehicle.

This work was dedicated to the development of a stimuli-responsive therapeutic hydrogel that aims to eliminate microtumor residues from surgically resected melanoma through the coupled effect of NIR light-induced photothermal therapy and heat-induced DOX release (Figure 1). To accomplish this, we functionalized GO as a thermo-responsive vehicle for DOX release and embedded it within a polymeric matrix of decellularized porcine small intestine submucosa methacrylate (SISMA) and chitosan methacrylate (ChiMA), yielding a photo-responsive material with tunable mechanical properties that is suitable for *in situ* deposition and room-temperature polymerization. All in all, we put forward a biomimetic and adhesive material with remarkable photothermal activity for the post-surgical treatment of resected malignant tumors. Furthermore, we studied heat and mass transport *in silico* via multiphysics simulations to evaluate the performance of the proposed chemo/photothermal therapy. The synthesized material underwent extensive physicochemical and biological characterization tests to evaluate its potential comprehensively, including mechanical response, microscopic imaging, methacrylate level, drug release *in vitro*, and biocompatibility.

## Materials and Methods

### Small Intestine Submucosa Decellularization

Porcine small intestines were decellularized following Sánchez-Palencia’s et al. (2014). Briefly, healthy segments of 10 cm were selected from the jejunum, hydrated in Type-II water, and stored at −20°C for 24 h to promote the perforation of cellular membranes by water crystals. To isolate the submucosa layer, the tunica mucosa, serosa, and muscularis layers were mechanically removed. The submucosa membranes were treated with a solution of hydrogen peroxide, sodium hypochlorite (PanReac AppliChem, Chicago, IL, United States), and type II water for 15 min under constant agitation at 190 RPM followed by washing with type II water and sterile 1X PBS (Sigma-Aldrich, St. Louis, MO, United States) to remove cellular and chemical remnants. Finally, washed membranes were dried at room temperature in a laminar flow hood overnight and subsequently pulverized by cryogenic grinding in a Freezer Mill (6,875 Freezer/Mill, SPEX SamplePrep, Metuchen, NJ, United States). Samples of decellularized SIS were then stored at 4°C until further use.

### Methacrylation of Small Intestine Submucosa and Chitosan

**FIGURE 2:**
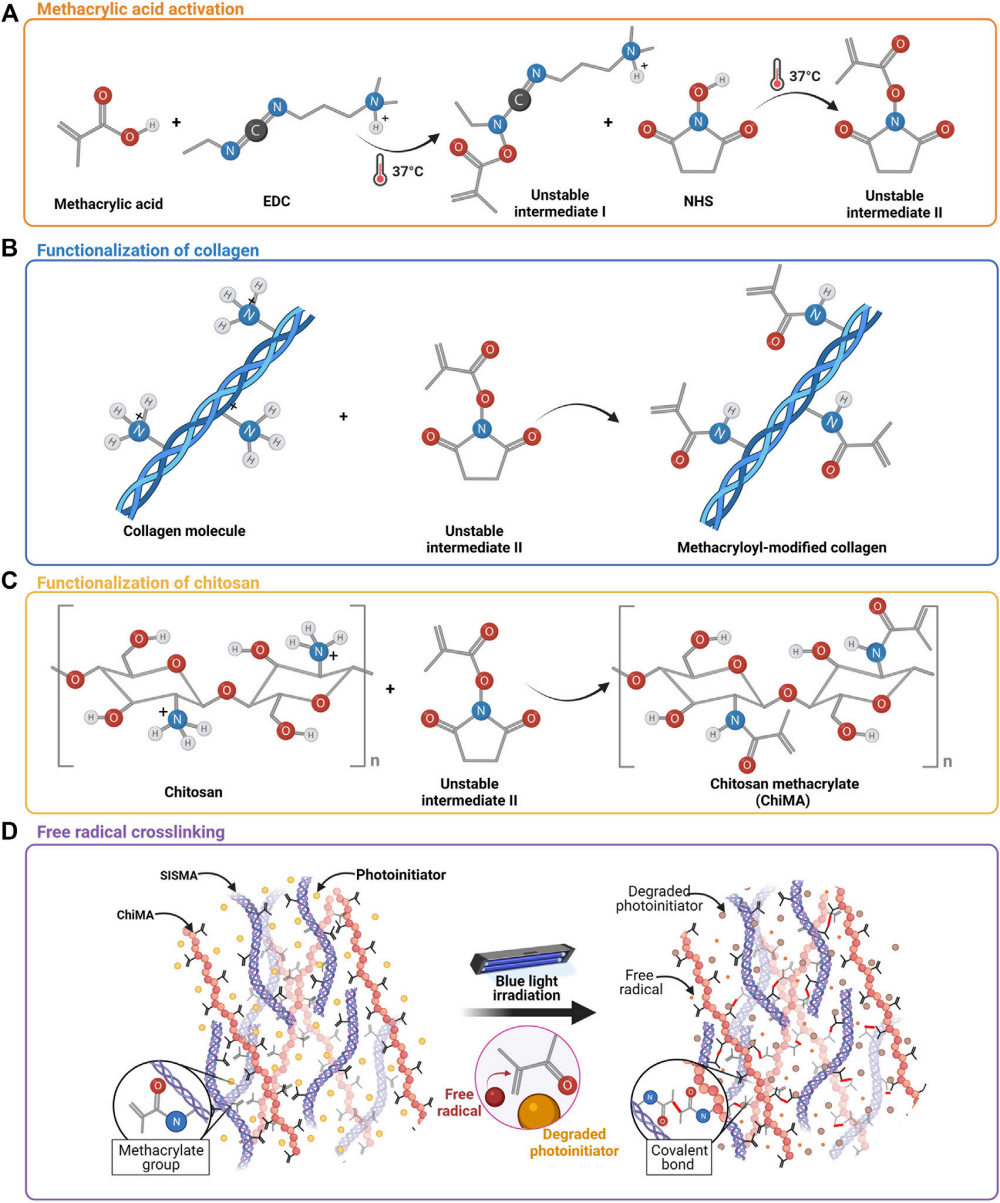
Methacrylation of SIS and chitosan. **(A)** Activation of methacrylic acid mediated by EDC and NHS. **(B)** Chemical conjugation of MA’s carboxyl groups to free primary amines of collagen in SIS. **(C)** Chemical conjugation of MA’s carboxyl groups into the free amines of the glucosamine units of chitosan. **(D)** Methacryloyl group destabilization and crosslinking are induced by the light-directed degradation of a photoinitiator.

MA’s carboxyl groups were covalently bound to the primary amines present in collagen strands of SIS through EDC/NHS-mediated activation, following the protocol by Rueda-Gensini et al. (2021) (Figures 2A,B). Pulverized SIS was solubilized at 4 mg/ml in acetic acid 0.5 M (PanReac AppliChem, Chicago, IL, United States) in the presence of pepsin 1 mg/ml (Sigma-Aldrich, St. Louis, MO, United States) for 48 h under vigorous magnetic stirring at 400 RPM. Meanwhile, MA, EDC, and NHS were mixed in 3 ml of DMF 99.8% (v/v) (Sigma-Aldrich, St. Louis, MO, United States) at a 1:20 M ratio with respect to the quantified free-amine groups in collagen and activated for 15 min at 37°C before their addition to the SIS solution. This mixture was then left to react for 24 h at 60°C under continuous magnetic stirring at 600 RPM. SISMA was dialyzed for 48 h against acetic acid 0.5 M and lyophilized for 48 h before sterilization with ethylene oxide.

Similarly, MA’s carboxyl groups were bound to the pendant primary amines of chitosan monomers through the same conjugation reaction described above, following the protocol reported by Céspedes-Valenzuela et al. (2021a) (Figures 2A,C). High-density chitosan (Sigma-Aldrich, St. Louis, MO, United States) was solubilized in acetic acid 0.17 M at 3.5 mg/ml for 10 min under magnetic stirring at 400 RPM. Meanwhile, MA, EDC, and NHS were mixed in 3 ml of DMF 99.8% (v/v) at a 1:2 M ratio with respect to the estimated free-amine groups and activated for 15 min at 37°C before their addition to the chitosan solution. This mixture was then left to react for 24 h at 60°C under continuous magnetic stirring at 600 RPM. ChiMA was dialyzed for 48 h against acetic acid 0.17 M and lyophilized for 48 h before sterilization with ethylene oxide.

### Synthesis of GO-DOX

GO was synthesized by the coupled exfoliation/oxidation of graphite, following Tour’s method (Marcano et al., 2010). Briefly, a mixture of 90 ml of sulfuric acid 98% (v/v) and 10 ml of phosphoric acid 85% (v/v) (PanReac AppliChem, Chicago, IL, United States) was slowly added to 0.75 g of graphite flakes (Graphene Supermarket, Ronkonkoma, NY, United States) and 4.5 g of potassium permanganate (PanReac AppliChem, Chicago, IL, United States) and left to react under constant magnetic stirring at 600 RPM and 50°C. After 12 h, 150 ml of type I water ice cubes and 3 ml of hydrogen peroxide 30% (w/w) were added to the formed viscous solution, thus inducing a color change from dark purple to gold yellow. The obtained GO was sonicated for 5 min with an amplitude of 38% and a frequency of 40 kHz and washed successively by filtration with polyester fiber, centrifugation at 4000 RPM for 4 h, and resuspension in a washing solution containing 50 ml of HCl 30% (v/v), 50 ml of ethanol 96% (v/v) (PanReac AppliChem, Chicago, IL, United States) and 50 ml of type I water. The final pellet was resuspended in type I water and lyophilized for 24 h.

**FIGURE 3:**
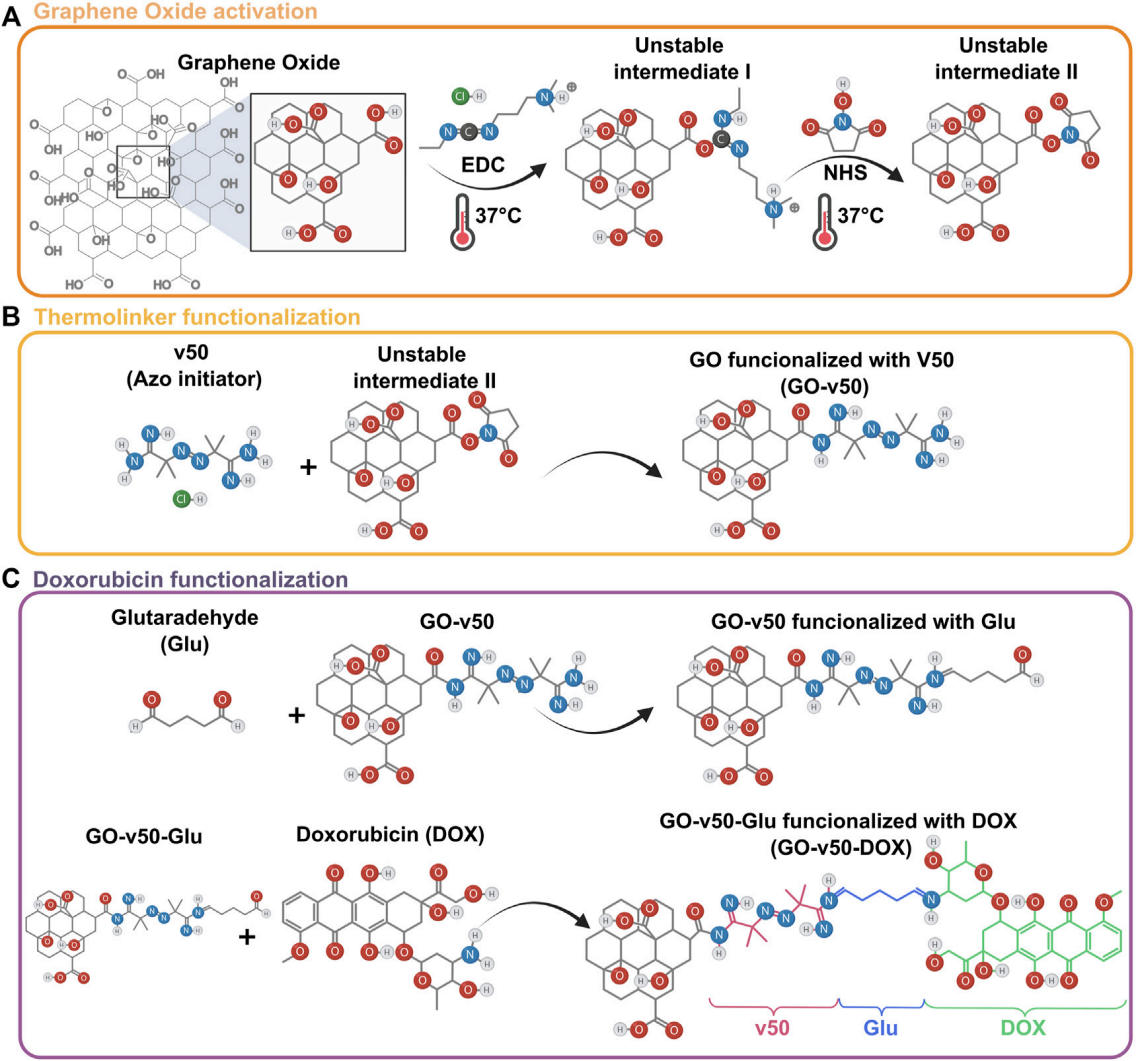
Loading of DOX into a GO vehicle. **(A)** Activation of graphene oxide (GO) mediated by EDC and NHS. **(B)** Chemical conjugation of the v50 thermo-linker to GO. **(C)** Conjugation of doxorubicin (DOX) to the GO-v50 nanoconjugate using glutaraldehyde as a crosslinker.

To convert GO into a thermoresponsive vehicle for DOX delivery, the nanomaterial was functionalized in two stages: linking v50 to GO, and linking DOX to v50 (Figure 3). GO and v50 (Fujifilm, Tokyo, Japan) were solubilized separately in type I water at a 4:1 mass ratio. Meanwhile, EDC and NHS were mixed in 3 ml of DMF 99.8 (v/v) at a 4:1 M ratio with respect to v50 and activated for 15 min at 37°C, after which the GO and v50 solutions were added and left to react for 24 h at room temperature under continuous magnetic stirring at 600 RPM. The obtained GO-v50 nanoconjugate was washed successively to remove excess reagents by centrifugation at 4000 RPM for 4 h. After 4 washing cycles, glutaraldehyde (Glu) (Sigma-Aldrich, St. (Sigma-Aldrich, St. Louis, MO, United States) was added to the purified GO-v50 at a 1:1 M ratio with respect to v50 and left under continuous magnetic stirring at 600 RPM for 1 h, while DOX (Sigma-Aldrich, St. Louis, MO, United States) was solubilized in type I water at a 1:1 M ratio with respect to v50. Pre-dissolved DOX was then dripped into the GO-v50-Glu solution and left under continuous magnetic stirring at 600 RPM for 24 h, followed by several washing cycles as described above. The obtained GO-v50-DOX nanoconjugate was lyophilized for 24 h and stored at 4°C until further use.

The proper synthesis and functionalization of GO were confirmed through FTIR, Raman spectroscopy, thermogravimetric analysis (TGA), transmission electron microscopy (TEM), and atomic force microscopy (AFM). FTIR was recorded in an A250 FTIR (Bruker, Germany) instrument for a spectral range of 4,000–400 cm^−1^, while Raman was recorded in an XploRA Confocal Raman Microscope (Horiba Scientific, Japan). TGA was recorded in a TG analyzer (TA Instruments, New Castle, DE, United States) using a temperature ramp of 25–600°C at a heating rate of 10°C/min, and under a nitrogen atmosphere. TEM imaging was conducted at 150,00X by a Tecnai F30 Microscope (Fei Company, Hillsboro, OR, United States), while AFM imaging proceeded in an MFP-3D-BIO AFM (Asylum Research, Santa Barbara, CA, United States) by employing AC240TS cantilevers (Oxford Instruments, Bognor Regis, United Kingdom) in AC mode.

### Hydrogel Preparation

Lyophilized SISMA and ChiMA samples were resuspended at 20 mg/ml or 40 mg/ml in acetic acid 0.02 M. In parallel, a working solution of DMEM (Gibco, Amarillo, TX, United States) supplemented with 10% (v/v) FBS (BioWest, Riverside, MO, United States), 0.1% (w/v) riboflavin, and Tris-HCl 0.1 M (Sigma-Aldrich, St. Louis, MO, United States) was prepared and adjusted to a pH of 8.5 with NaOH 5 M (PanReac AppliChem, Chicago, IL, United States). Resuspended SISMA and ChiMA solutions were mixed at a 1:1 volume ratio with the working solution to prepare two formulations: SISMA 1%/ChiMA 1% (S1C1) and SISMA 2%/ChiMA 1% (S2C1). The most promising formulation was laden with the GO-v50-DOX nanoconjugate and ascorbic acid (Sigma-Aldrich, St. Louis, MO, United States), which were pre-dispersed in the working solution at 1 mg/ml and 4 mg/ml (S2C1GO) (Céspedes-Valenzuela et al., 2021b; Rueda-Gensini et al., 2021).

Hydrogels were loaded into 3 ml syringes and manually ejected through a 21-gauge needle to assess their potential to form continuous filaments during extrusion. After deposition, samples were irradiated for 10 min at 62 mW/cm^2^ with blue light (420–460 nm) to induce covalent crosslinking between the methacryloyl groups of SISMA and ChiMA (Serna et al., 2019).

### Rheological Evaluation

Experiments to evaluate the rheological behavior of formulations were performed in a Discovery Series Hybrid Rheometer-1 (TA Instruments, New Castle, DE, United States) using a parallel plate geometry with a 20 mm gap. Changes in both the storage (G’) and loss (G”) moduli of photocrosslinked samples were assessed through flow, frequency, time, and temperature sweeps. The flow sweep experiments were conducted from 0.01 to 100 Hz at 1% strain at room temperature. Frequency sweep experiments were performed between 0.01 and 100 rad/s at room temperature. Last, coupled time/temperature sweep experiments were carried out under oscillatory mode and a constant strain of 1% and 10 rad/s with a temperature ramp of 20°C/min between 15 and 37°C. Shear-thinning properties of the formulations were estimated by fitting the viscosity (η) versus shear rate (γ) plot to the power-law model (Eq. 1):

$$\eta =K\gamma^{n-1}$$ (1)

### Dispersion of GO-DOX

To evaluate the dispersion of GO and DOX within the nanocomposite hydrogels, the materials’ self-fluorescence was imaged through confocal microscopy at 405 and 480 nm, respectively. Hydrogel samples were imaged at ×20 magnification with an FV1000 Confocal Microscope (Olympus, Tokyo, Japan), and particle count and area were estimated with the aid of the ImageJ^®^ software. To assess the spatial distribution of GO sheets within the hydrogel, a Z-stack reconstruction was performed by capturing images at different depth positions (Rueda-Gensini et al., 2021).

### Morphological Structure of the Hydrogels

The polymeric microstructure of the nanocomposite hydrogels was observed before and after photocrosslinking via scanning electron microscopy (SEM). S2C1GO samples were imaged at ×500 and ×1500 magnifications in a JSM 6490-LV microscope (JEOL, Tokyo, Japan) under vacuum conditions and a 20 kV accelerating voltage. Pore size distribution was determined with the aid of the ImageJ^®^ software.

### Qualitative Adhesion

The adhesion potential of the nanocomposite hydrogels was qualitatively assessed by sealing a cut wound under physiological conditions. Frozen porcine skin samples were thawed in warm water and a 1.5 cm long, 0.5 cm deep linear wound was cut open with a surgical scalpel. 500 µL of each hydrogel were extruded and photocrosslinked over the wound. Treated samples were incubated in PBS 1X at 37°C and checked daily until the wound seal was broken.

### Texture Analysis

The firmness of the hydrogels was evaluated with a TA.HDplusC Texture Analyzer (Stable Micro Systems, Godalming, United Kingdom) before and after sample irradiation. Nanocomposite hydrogels were molded into 20 mm diameter and 25 mm height cylinder-shaped constructs. This test measured the compression force at a 1.0 mm/s speed and 15 mm penetration length using a 10 mm cylindrical probe (Patarroyo et al., 2021).

### Hemolysis and Platelet Aggregation

To assess the hemolytic activity of the nanoconjugates and nanocomposite hydrogels, blood samples were collected in EDTA tubes and centrifuged at 1800 RPM for 5 min to separate the plasma and replace it with PBS. This procedure was repeated until a purified erythrocyte precipitate was obtained. 100 ml of a 10% (v/v) PBS-diluted erythrocyte solution were mixed with 100 ml of each extract and incubated for 1 h at 37°C. The samples were then centrifuged at 3000 RPM for 5 min and 100 ml from the supernatant were seeded in triplicate in a 96-well microplate and read at 450 nm in a Multiskan FC Microplate reader (Thermo Fisher Scientific, Waltham, MA, United States). Triton 100-X and PBS 1X were used as positive and negative controls. The percentage of hemolytic activity was estimated by the following equation (Eq. 2):

$$\mathrm{Platelet}\;\mathrm{aggregation}\left(\%\right)=\frac{\mathrm{Ab}s_{s}-\mathrm{Ab}s_{\left(-\right)}}{\mathrm{Ab}s_{\left(+\right)}-\mathrm{Ab}s_{\left(-\right)}}\times 100\%$$ (2)

For platelet aggregation, blood samples were collected in sodium citrate tubes and centrifuged at 1000 RPM for 15 min to retrieve the platelet-rich plasma (PRP). 100 ml of each extract were seeded by triplicate with 100 ml of PRP in a 96-well microplate and left under constant agitation for 1 h. The unaggregated platelets in the supernatant (100 ml) were retrieved, seeded in another microplate with 10 ml of triton 100-X, and left under agitation for 15 min. This microplate was then centrifuged at 1000 RPM for 15 min and 50 ml of each well were retrieved and seeded in another microplate. Finally, 50 ml of LDH reagent were added to each well and the absorbance was read at 493 nm in a Multiskan FC Microplate reader (Thermo Fisher Scientific, Waltham, MA, United States). Platelet aggregation was estimated by comparing absorbances to an LDH calibration curve (Supplementary Figure S1).

### Cytotoxicity

The cytotoxicity of the nanoconjugate and nanocomposite hydrogels was determined by an indirect contact assay with MTT (Sigma-Aldrich, St. Louis, MO, United States). First, hydrogel samples were mixed with FBS-free DMEM at 25% (v/v) and incubated for 4 h at 37°C. Extracts for the assay were obtained by retrieving the supernatant of the centrifuged mixtures and preparing dilutions at 25, 12.5, 6.25, 3.13, and 1.56% (v/v). In parallel, 100 µl of A-375 cells (American Type Culture Collection, Manassas, VA, United States) were seeded in a 96-well culture plate at a density of 1 × 106 cells/mL and incubated at 37°C, 5% CO2 for 24 h. Culture media was then removed and replaced with FBS-free DMEM. For each of the dilutions, 100 µl of the hydrogel extracts were added in triplicate to cell-seeded wells, and the culture plate was incubated at 37°C, 5% CO2 for another 48 h. FBS-free DMEM and DMSO (Sigma-Aldrich, St. Louis, MO, United States) were added instead of the extracts as negative and positive controls, respectively. 10 μl of MTT (5 mg/ml) were added to each well, and cells were incubated at 37°C, 5% CO2 for 2 h. Culture medium was removed, and 100 μl of DMSO was added to dilute formazan crystals. Absorbance was read at 595 nm in a Multiskan FC Microplate reader (Thermo Fisher Scientific, Waltham, MA, United States) (Lopez-Barbosa et al., 2019).

### *In silico* Analysis of the Chemo/Photothermal Therapy

The photothermal treatment of malignant cutaneous melanoma was modeled in two steps: photo- and thermo-responsive composite hydrogels localized heat transfer and mass diffusion post-thermo-linker breakup for the controlled delivery of DOX. Simulations were carried out via the Finite Element Method (FEM) with the aid of the COMSOL Multiphysics^®^ software. Following the development of the model, an optimization function was defined to estimate the optimal heating time depending on the main treatment parameters.

The Bioheat Transfer physics module was used to study Near Infrared light (NIR) stimulation. Hydrogel and biological tissue (skin multilayer model) heating were described by the following governing equation (Pennes, 1948) (Eq. 3):

$$\rho C_{p}\frac{\partial T}{\partial t}=k\left[\frac{1}{r}\frac{\partial }{\partial r}\left(r\frac{\partial T}{\partial r}\right)+\frac{\partial^{2}T}{\partial z^{2}}\right]+\rho_{b}C_{p,b}V_{b}^{v}\left(T_{b}-T\right)+Q_{m}+Q_{\mathrm{NIR}}$$ (3)

where ρ is the tissue density, *C*_*p*_ is the tissue-specific heat, k is the tissue thermal conductivity, *ρ*_*b*_ *C*_*p,b*_ *V*_*b*_^*v*^ is the bioheat transport by blood perfusion, *Q*_*m*_ metabolic heat generation rate of the tissue, and *Q*_*NIR*_ is the heat generated by NIR stimulation, defined by an exponential decay function with respect to depth (i.e., the *z*-axis of the simulation’s coordinate system) described in Eq. 4:

$$Q_{\mathrm{NIR}}=\mathrm{E\alpha}I_{0}e^{-\mathrm{\alpha z}}$$ (4)

where E is the photothermal conversion efficiency coefficient of rGO, α is the specific absorption rate, and *I*_*0*_ is the NIR source intensity.

DOX controlled delivery throughout the tumor and the surrounding tissue after the thermo-linker breakup was simulated with the aid of the Transport of Diluted Species physics module. Mass transfer was modeled as a transient process with the following governing equation (Eq. 5):

$$\frac{\partial C_{D}}{\partial t}+u_{r}\frac{\partial C_{D}}{\partial r}+u_{z}\frac{\partial C_{D}}{\partial z}=\{\begin{array}{cc} 0, & x<T=45{}^{\circ}C\\ D_{D,\mathrm{tissue}}\left[\frac{1}{r}\left(\frac{\partial }{\partial r}r\frac{\partial C_{D}}{\partial r}\right)+\frac{\partial^{2}C_{D}}{\partial z^{2}}\right], & x\geq T=45{}^{\circ}C \end{array}$$ (5)

where *C*_*D*_ is the doxorubicin concentration, *D*_*D*_, tissue is the diffusivity of DOX through the tissue, and ∂r and ∂z are the radial and axial components of velocity.

Likewise, the degree of tissue injury, Ω, is computed to study the behavior of healthy tissue under heat treatment and guarantee the specificity of tumor damage. Thermal damage is described by Arrhenius kinetics following the equation presented by Henriques and Moritz (1947) (Eq. 6):

$$\frac{\partial \Omega }{\partial t}=Ae^{-\frac{\Delta E}{\mathrm{RT}}}$$ (6)

where A is a pre-exponential frequency factor, ∆E is defined as the activation energy and R is the universal gas constant.

**FIGURE 4:**
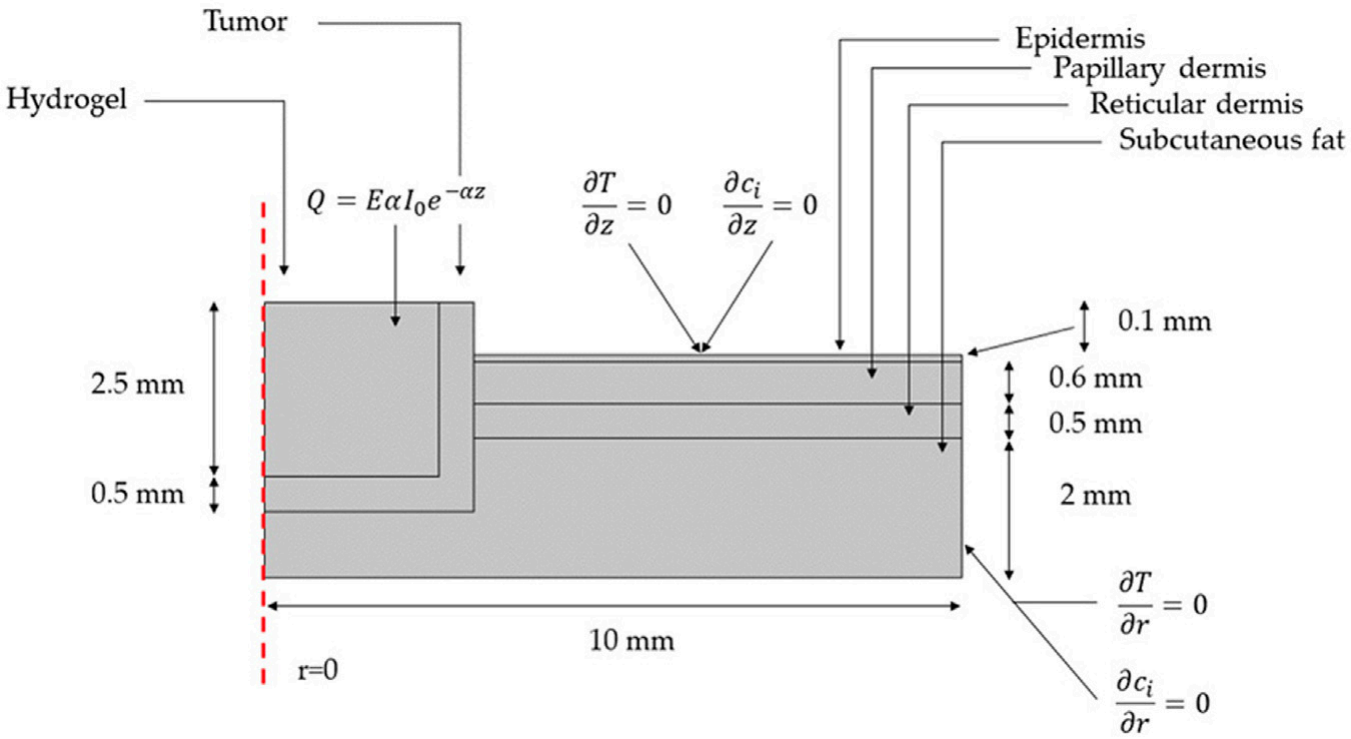
Geometry and boundary conditions for the NIR-induced heating and doxorubicin-controlled release model involving tumor residual cells after the removal of malignant cutaneous melanoma and the surrounding skin layers of healthy tissue.

Figure 4 shows a 2D axisymmetric geometry for a multilayer skin model with its corresponding dimensions. Computational domains are presented for the hydrogel, the remnants of cancerous tissue after tumor extraction, and the different layers of surrounding healthy tissue (i.e., epidermis, papillary dermis, reticular dermis, and subcutaneous fat). As boundary conditions, no convective heat flux was applied over the computational domain. Neither heat flux nor mass transfer was considered beyond the modeled tissue. Table 1 summarizes the parameters and domain materials used for both the heat Bioheat Transfer and Transport of Diluted Species models.

**TABLE 1:** Parameters and domain materials used for the heat Bioheat Transfer and Transport of Diluted Species models.

Parameter	Value
NIR source intensisty, I0	70,000 W/m^2^
Speficif absorption rate, α	40 m^−1^
Thermo-linker breakup temperature, Tl	45°C
Tumor necrosis temperature, Tn	49°C
Skin damage temperature, Ts	44°C
Thermal conductivity, k	
Epidermis	0.235 W/mK
Papillary dermis	0.445 W/mK
Reticular dermis	0.445 W/mK
Subcutaneous fat	0.185 W/mK
Clark V Melanoma	0.588 W/mK
Hydrogel	470 W/mK
Density, ρ	
Epidermis	1,200 kg/m^3^
Papillary dermis	1,200 kg/m^3^
Reticular dermis	1,200 kg/m^3^
Subcutaneous fat	850 kg/m^3^
Clark V Melanoma	1,030 kg/m^3^
Hydrogel	1,000 kg/m^3^
Specific heat, Cp	
Epidermis	3600 J/kgK
Papillary dermis	3800 J/kgK
Reticular dermis	3800 J/kgK
Subcutaneous fat	2300 J/kgK
Clark V Melanoma	3852 J/kgK
Hydrogel	4000 J/kgK
Metabolic heat rate, Qm	
Epidermis	0 W/m^3^
Papillary dermis	627.8 W/m^3^
Reticular dermis	627.8 W/m^3^
Subcutaneous fat	3767 W/m^3^
Clark V Melanoma	65,400 W/m^3^
Photothermal conversion coefficient, E	0.58
Bloor perfusion rate, ωb	0.0036 s^−1^
Pre-exponential frequency factor, A	7.39∙1039 s^−1^
Activation energy, ∆E	2.577∙105 J/mol
Doxorubicin diffusivity, DD	4∙10–10 m^2^/s

To optimize the heating time to maximize tumor damage while minimizing surrounding healthy tissue damage an objective function J was defined as (Eq. 7):

$$J=\sum_{i}f_{T}\left(T_{i}\right)+\sum_{j}f_{N}\left(T_{j}\right)$$ (7)

where:

$$f_{T}\left(T_{i}\right)=\{\begin{array}{cc} T-T_{n}, & T>T_{n}\\ 0, & T_{l}\leq T\leq T_{n}\\ T_{l}-T, & T<T_{l} \end{array}$$ (8)

$$f_{N}\left(T_{j}\right)=\{\begin{array}{cc} 0, & T\leq T_{s}\\ T-T_{s}, & T>T_{s} \end{array}$$ (9)

This function sums up the objective function at all *i* temperatures within the tumor tissue, and all *j* temperatures within the normal tissue. When the temperature of the healthy tissue is below the limit for significant damage ( $T_{s}$ = 44°C), and the tumor is heated up to the ideal temperature range for DOX release and necrosis (45°C–49°C), the function will be optimized (i.e., decreased) promoting tumor elimination without compromising surrounding healthy tissues. $T_{n}$ was chosen as the upper temperature threshold for the thermo-linker breakup and consequently, DOX release.

### Photothermal Transduction of rGO and Release of DOX

The chemo/photothermal effect of the GO-DOX-laden nanocomposite hydrogels was evaluated by subjecting samples to different light and heat stimuli. 300 ml of the nanocomposite hydrogel were extruded into cylindrical constructs, crosslinked for 2 min at 62 mW/cm^2^ by irradiation with blue light (420–460 nm), and incubated at 37°C for 8 h to reduce GO into rGO. Nanocomposite hydrogels were then irradiated with a High Power 808 nm NIR Dot Laser Module (MXTLASER, Zhongshan, China) for 3 min at 7 W/cm^2^ and left to rest 30 min in an incubator at 37°C for 3 consecutive rounds. To assess the photothermal conversion of rGO, temperature changes were measured with a FLIR E60 Advanced Thermographic Camera (FLIR Systems, Wilsonville, OR, United States) during each heating cycle. Similarly, supernatants were recovered after each therapy cycle to measure the released DOX aided by a 0239D-2219 FluoroMax plus C spectrofluorometer (Horiba, Miyanohihashi, Japan). Pristine hydrogels and therapies in the absence of NIR irradiation were used as transduction and release controls, respectively.

### Statistical Analysis

All experimental data were collected in triplicate, for which mean and standard deviation were calculated. Statistical analysis was performed using two-way ANOVA and Tukey’s test for pairwise comparison in the software GraphPad Prism^®^ (GraphPad Software Inc., San Diego, CA, United States). *p* < 0.05 was considered statistically significant.

## Results and Discussion

### Chemical Functionalization of SIS and Chitosan

The chemical conjugation of methacryloyl groups to SIS and chitosan was achieved by following the synthesis protocols reported by Rueda-Gensini et al. (2021) and Céspedes-Valenzuela et al. (2021a), which aimed to induce enhanced methacrylation levels for these polymers. This modification was confirmed by changes in the FTIR spectra of SIS and chitosan (Supplementary Figure S2). In this regard, the presence of peaks at 1,680 cm^−1^ and 670 cm^−1^ corresponded to the C=O stretching and C=C bending vibrations present in the amide-bound methacryloyl groups (Sharma et al., 2014; Krylova and Dukštienė, 2019).

### Synthesis of Graphene Oxide

**FIGURE 5:**
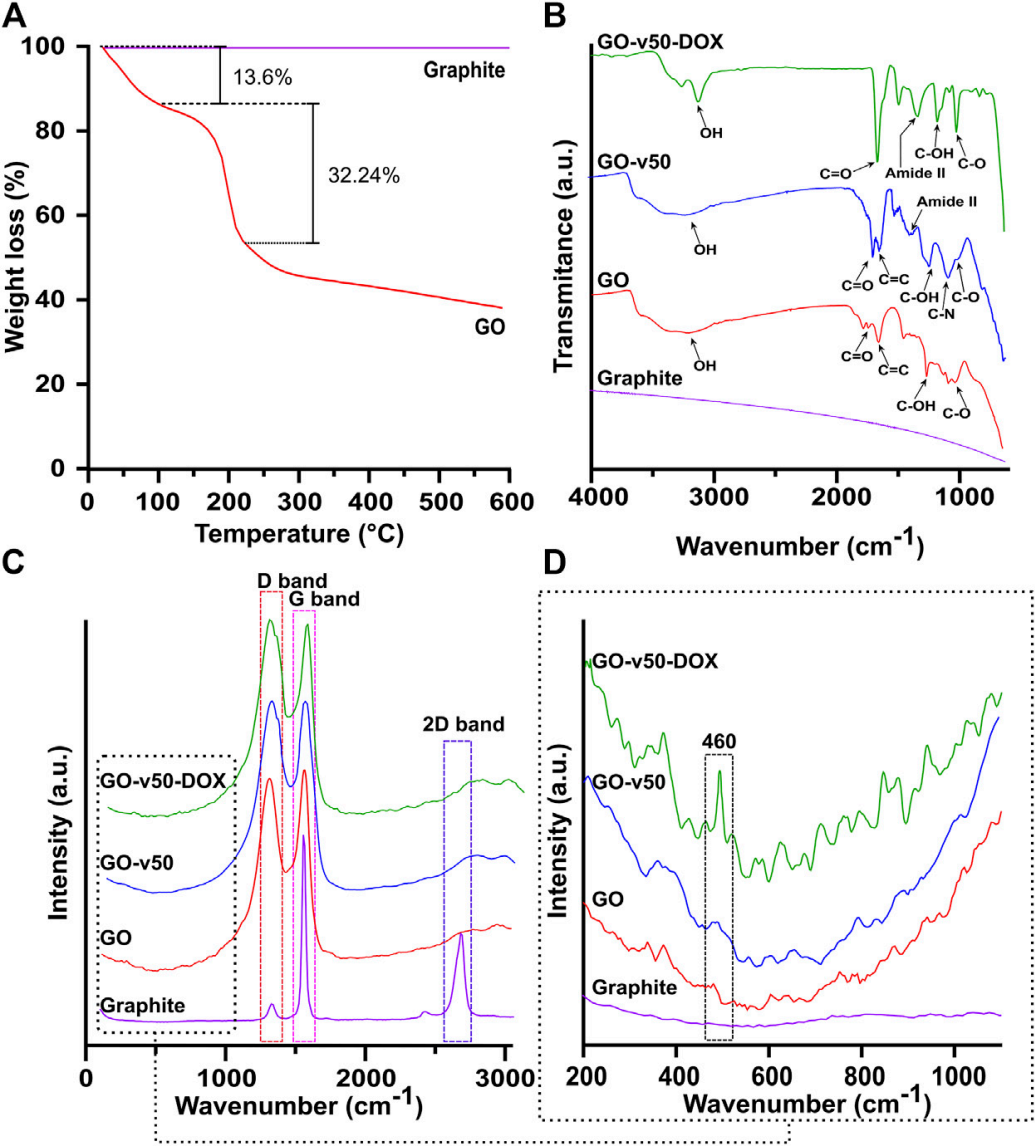
Thermal and spectroscopic analyses of pristine and modified GO. **(A)** TGA of GO. **(B)** FTIR spectra of graphite, pristine GO, GO-v50 nanoconjugate, and GO-v50-DOX nanoconjugate. **(C,D)** Raman spectra of graphite, pristine GO, GO-v50 nanoconjugate, and GO-v50-DOX nanoconjugate.

**FIGURE 6:**
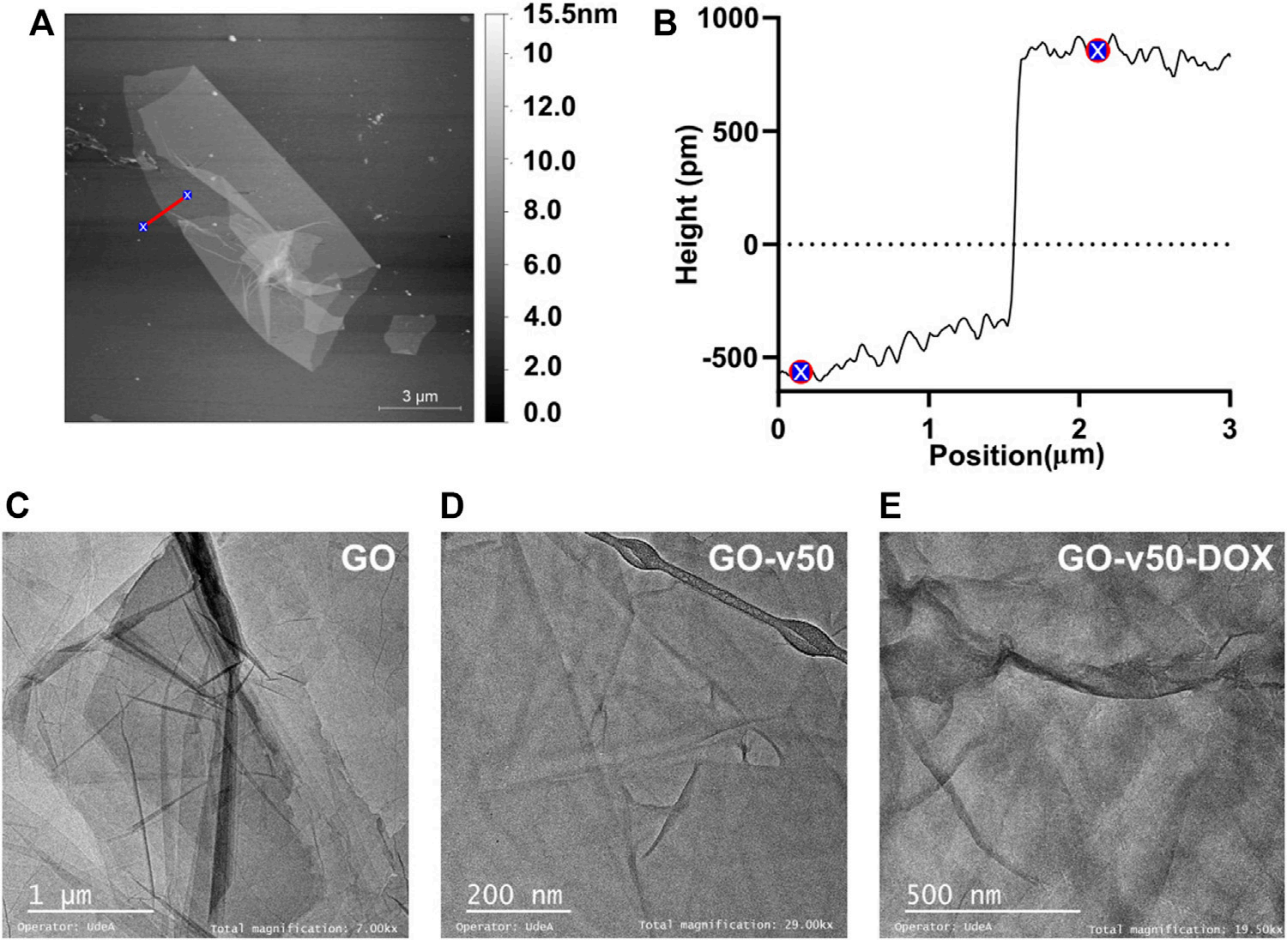
Microscopy of pristine and modified GO. **(A)** AFM images of GO, **(B)** Sheet height of pristine GO, and TEM images of **(C)** pristine GO, **(D)** GO-v50 nanoconjugate, and **(E)** GO-v50-DOX nanoconjugate.

The correct synthesis and functionalization of GO were confirmed by the TGA, FTIR, Raman spectroscopy, AFM, and TEM analyses as shown in Figures 5, 6. The TGA thermogram of GO (Figure 5A) presents three noticeable weight losses. A first weight loss of 13.6% from room temperature to 100°C can be attributed to the evaporation of bound water. A second weight loss of 32.24% between 100–200°C is most likely related to labile functional groups. A final loss between 200–600°C can be attributed to the removal of oxygen-rich species, further confirming GO’s high level of oxidation (Stankovich et al., 2007; Feng et al., 2013).

The FTIR spectra (Figure 5B) confirm the correct oxidation of graphite, as GO exhibits multiple peaks related to oxygen-rich functional groups (Yoo et al., 2014; Strankowski et al., 2016). Peaks at 3,392 cm^−1^ and 1,226 cm^−1^ correspond to O-H and C-OH stretching vibrations, while peaks at 1740 cm^−1^ and 1,050 cm^−1^ represent C=O stretching and C-O bending vibrations, respectively. The peak at 1,620 cm^−1^ can be attributed to C=C aromatic stretching vibration. The absence of peaks near 1,250 cm^−1^ confirms that the nanoconjugate was not partially reduced into rGO (Marcano et al., 2010; Krishnamoorthy et al., 2013). Conjugation of v50 to GO led to the reduction of hydroxyls of carboxyl groups to form amide II bonds with one of the linker’s N-terminals, thereby yielding noticeable increments in the amide II peak at 1,400 cm^−1^, relative to C-OH stretching vibration. The primary amines present at the unbound end of v50 most likely led to a peak at 1,120 cm^−1^, while azo bonds and imidine groups were not observable in the FTIR spectrum. Furthermore, the spectrum of the GO-v50-DOX nanoconjugate presents a decrease in C-N groups, as the free amine terminal of v50 turns into an imine group by the conjugation of glutaraldehyde. Peaks involving C-O, C-OH, and C=O experience a substantial increase, as newly bound DOX start to prevail on the surface of GO.

The Raman spectrum (Figures 5C,D) of graphite presents strong G and 2D bands at 1,570.8 cm^−1^ and 2,705.1 cm^−1^, while the spectrum of GO shows strong G and D bands at 1,588.9 cm^−1^ and 1,345.8 cm^−1^ (Eda and Chhowalla, 2010; Krishnamoorthy et al., 2011). The G band, which relates to the sp2 hybridization in carbon, most likely shifted to 1,588.9 cm^−1^ due to the oxidation of graphite (Krishnamoorthy et al., 2011). Similarly, the noticeable growth of the D band, which results from vacancies or dislocations that disrupt sp2 carbon layers, suggests the transition of some carbon atoms in graphene to an sp3 hybridization to accept functional groups (Muzyka et al., 2018). The conjugation of v50 to GO can be evidenced in the appearance of a small shoulder in the D band’s descent, which arises from a superimposed band centered at 1,368 cm^−1^ that correlates with the azo bond (N=N) from the linker’s structure (Çatikkaş, 2017). Furthermore, the conjugation of DOX can be observed in a small but distinguishable peak around 460 cm^−1^ that corresponds to C-O bonds (Farhane et al., 2015). Similarly, the XPS survey spectra (Supplementary Figure S3) and elemental composition (Table 2) confirm the increase of nitrogen due to the functionalization with the linker and DOX. Additionally, a decrease in the C1s area and binding energy in the GO-v50 spectrum is most likely due to the breakdown of the graphene resonance by its functionalization. The same band increases in the GO-DOX spectrum, which confirms the presence of C=O, C-O-, and C-O-C functional groups that are predominant in the conjugated DOX.

**TABLE 2:** Surface chemical analysis of the GO, GO-v50 and GO-v50-DOX nanocomposite samples. C1s, N1s and O1s decomposed peaks and related binding energies (BE), FHWM and area values are also shown.

Sample	Peak	EB (eV)	FHWM	Area
GO	C1s	284.60	1.99	1,236
		285.82	1.80	2,250
		287.72	1.56	3,731
		289.36	1.33	220
	O1s	531.42	1.63	1,509
		532.57	1.50	4,428
		533.45	1.48	4,492
GO-v50	C1s	284.60	2.30	592
		285.89	1.95	1,482
		287.81	1.55	911
		288.52	1.88	506
	N1s	399.23	1.50	57
		400.66	1.50	187
		402.05	1.50	109
	O1s	531.36	2.11	740
		532.46	1.49	1,446
		533.57	1.60	2092
GO-v50-DOX	C1s	284.60	1.60	600
		285.88	1.60	1,349
		287.77	1.60	2002
		289.13	1.62	496
	N1s	399.55	1.50	89
		400.91	1.50	340
		402.51	1.50	134
	O1s	531.68	2.35	1,468
		533.03	2.08	3,574
		533.91	1.82	1,601

AFM images of GO (Figure 6A) show a sheet-like morphology with different levels of stacking, which likely occur by aggregation or self-assembly during lyophilization. As evidenced in Figure 6B, the average sheet height corresponded to 0.8 nm, which is consistent with previous reports (Feng et al., 2013; Krishnamoorthy et al., 2013). Similarly, the TEM micrograph of GO nanosheets (Figures 6C–E) exhibits its characteristic flake-like structure (Song et al., 2012). Different levels of opacity in the image reveal a non-uniform stacking of multiple layers of GO; higher transparency indicates thinner films, while darker regions indicate larger stacking (Stobinski et al., 2014). With the advance of GO’s functionalization (i.e., GO, GO-v50 and GO-v50-DOX), the sheets become slightly more wrinkled due to attraction forces between newly introduced functional groups. Nevertheless, these minor conformational changes do not compromise the nanomaterial’s integrity or performance.

### Rheological Evaluation

**FIGURE 7:**
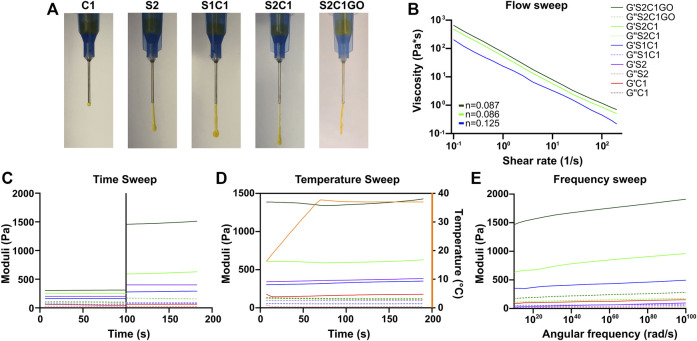
Rheology of the ChiMA 1% (C1), SISMA 2% (S2), SISMA 1%/ChiMA 1% (S1C1), SISMA 2%/ChiMA 1% (S2C1), and SISMA 2%/ChiMA 1%/graphene oxide (S2C1GO) hydrogels. **(A)** Manual injection of the hydrogels through a 21-gauge needle. **(B)** Flow sweeps of photocrosslinked samples. **(C)** Time sweeps before and after blue-light photocrosslinking. **(D)** Temperature sweeps of photocrosslinked samples between 5 and 37°C. **(E)** Frequency sweep of photocrosslinked samples.

Only two nanocomposite hydrogels (S1C1 and S2C1) were successfully prepared following the stated approach, as formulations with higher ChiMA content lacked homogeneity because alkaline environments tend to promote the formation of insoluble clusters (Shen et al., 2020). SISMA 2% (S2) and ChiMA 1% (C1) hydrogels were prepared as controls for rheology. All the tested hydrogels were able to form continuous filaments when extruded through a 21-gauge needle except for C1, which dripped ununiformly due to insufficient consistency (Figure 7A). Because of its superior integrity and overall performance during extrusion, S2C1 was embedded with GO (S2C1GO) and characterized with the rest of the hydrogels. The addition of GO into the hydrogel seemed to considerably improve its cohesion and integrity, as it enabled easier handling during extrusion and led to a more continuous and stable filament formation during manual extrusion (Figure 7A).

Figures 7B–E present the changes in the storage modulus (G′) and loss modulus (G″) of the hydrogels when subjected to different stimuli. The flow, temperature, and frequency experiments were performed over irradiated samples, as exposure to blue light (Figure 7C) significantly improved both moduli, favoring mechanical stability and a solid-like behavior (Coussot, 2017; Rueda-Gensini et al., 2021). The flow sweep (Figure 7B) shows that the hydrogels exhibit lower viscosity at higher shear rates, a characteristic behavior of pseudoplastic fluids. This shear-thinning behavior can be further confirmed by the power-law fittings shown in Supplementary Figure S4, which yield values of n < 1 for all the three evaluated formulations (Coussot, 2017; Paxton et al., 2017). Moreover, temperature increments between 15 and 37°C (Figure 7D) cause a slight increase in both G′ and G″, implying that more energy is required for deformation and thus suggesting superior crosslinking, which could be attributed to the gelling behavior of collagen (Sandolo et al., 2009; Zhang et al., 2012). However, these temperature-induced changes in the moduli are non-statistically significant (*p* > 0.9999). Furthermore, the frequency sweep (Figure 7E) shows that both moduli are frequency-dependent, as G’ and G” increase with angular frequency.

In general, all hydrogels present a higher G′ and a lower G″, confirming the material’s potential to store deformation energy with small dissipation from internal friction (Coussot, 2017). The C1 control yields insufficient G′ and G″ levels, suggesting its inability to maintain a mechanically stable polymer network, while the S2 control presents desirable values for both moduli and adequate response to stimuli. In S1C1, the poor performance of C1 partially overshadows the potential of S2, thus yielding lower moduli. In contrast, S2C1 exhibits a superior performance, which was further improved by the addition of GO (Jafarigol et al., 2020).

### GO Dispersion

**FIGURE 8:**
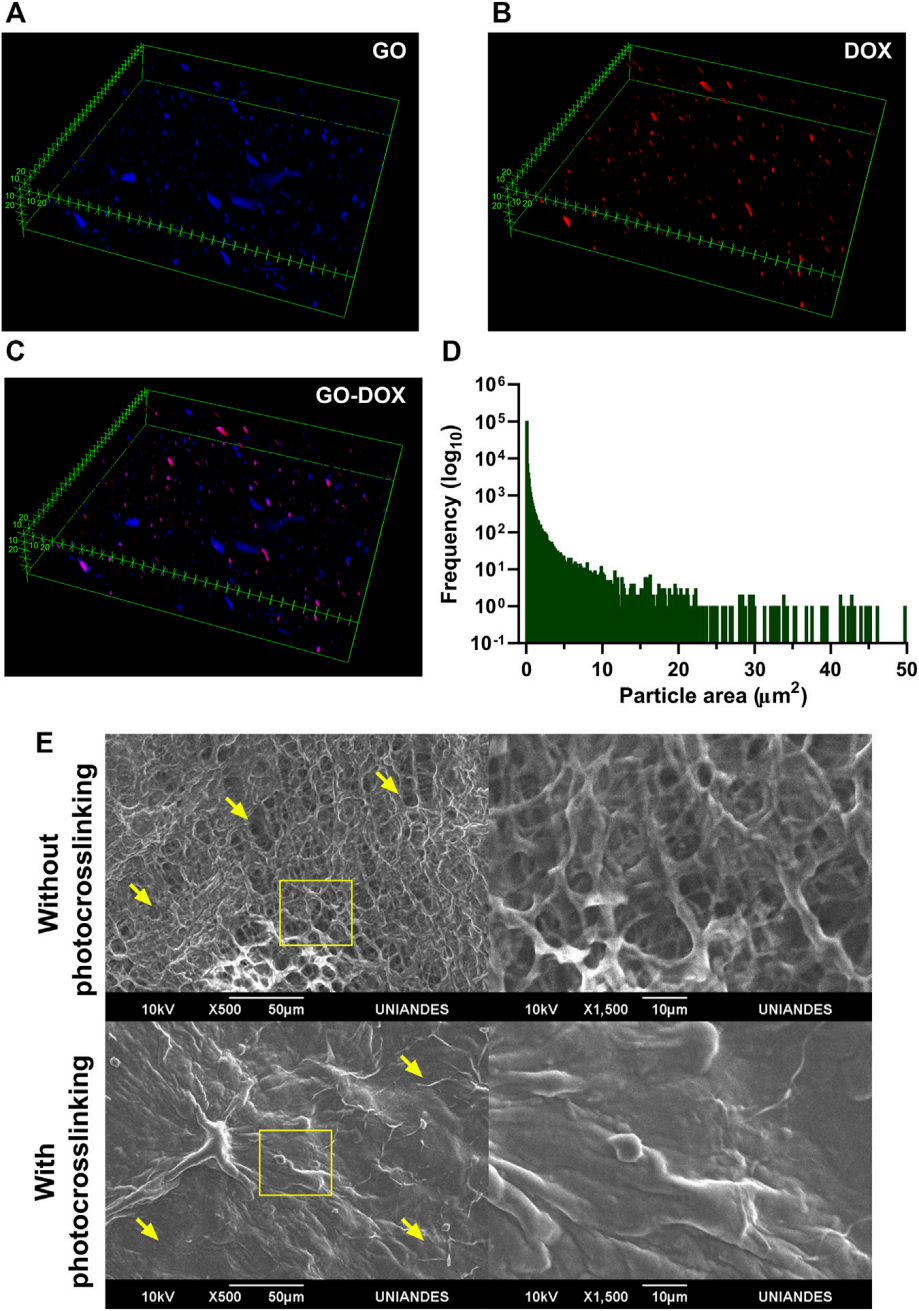
Dispersion of GO-DOX nanoconjugates and morphological features of the nanocomposite hydrogel. Dispersion of **(A)** pristine GO, **(B)** DOX, and **(C)** GO-DOX nanoconjugates in the SISMA/ChiMA matrix. **(D)** Particle size distribution of pristine GO, seen as a right tail distribution centered at 0.096 µm2 **(E)** SEM images of the microporous structure of the SISMA 2%/ChiMA 1%/graphene oxide (S2C1GO) formulation prior to and after photocrosslinking.

Dispersion of GO and DOX in the hydrogel matrix was observed in a z-stack reconstruction from confocal microscopy images. To avoid agglomeration, GO was mixed with FBS-supplemented DMEM before its addition to the S2C1 pregel, yielding the homogeneous dispersion presented in Figures 8C,D (Rueda-Gensini et al., 2021). This particle area distribution can be seen as a right tail distribution centered at 0.096 µm^2^ and with agglomerations as large as 348.450 µm^2^. Moreover, DOX’s self-fluorescence at 480 nm was imaged in Figure 8C. A careful inspection of the distributions shown in Figures 8A–C strongly suggests that unbound DOX is rarely found in the hydrogel.

Similarly, the morphology of the polymeric matrix in the S2C1GO nanocomposite hydrogel was imaged by SEM before and after photocrosslinking (Figure 8E). Under blue light irradiation, riboflavin degrades and generates free radicals that destabilize the alkene bonds in the methacryloyl groups of SISMA and ChiMA, thus inducing covalent crosslinking between adjacent strands and causing the microstructure to collapse (Céspedes-Valenzuela et al., 2021b). Changes in the porous structure are visually perceptible, as pore diameter varies between 2.1 and 27.2 µm with a mean of 11.7 µm for non-irradiated samples, while for irradiated samples, the material exhibits a collapsed structure with no measurable pores. Crosslinked samples also exhibit a rough and uneven microstructure, which can be attributed to the formation of imine bonds between GO and free amine radicals present in the collagen or chitosan strands (Sahraei and Ghaemy, 2017; Tohamy et al., 2020; Patarroyo et al., 2021).

### Adhesion and Texture Analyses

**FIGURE 9:**
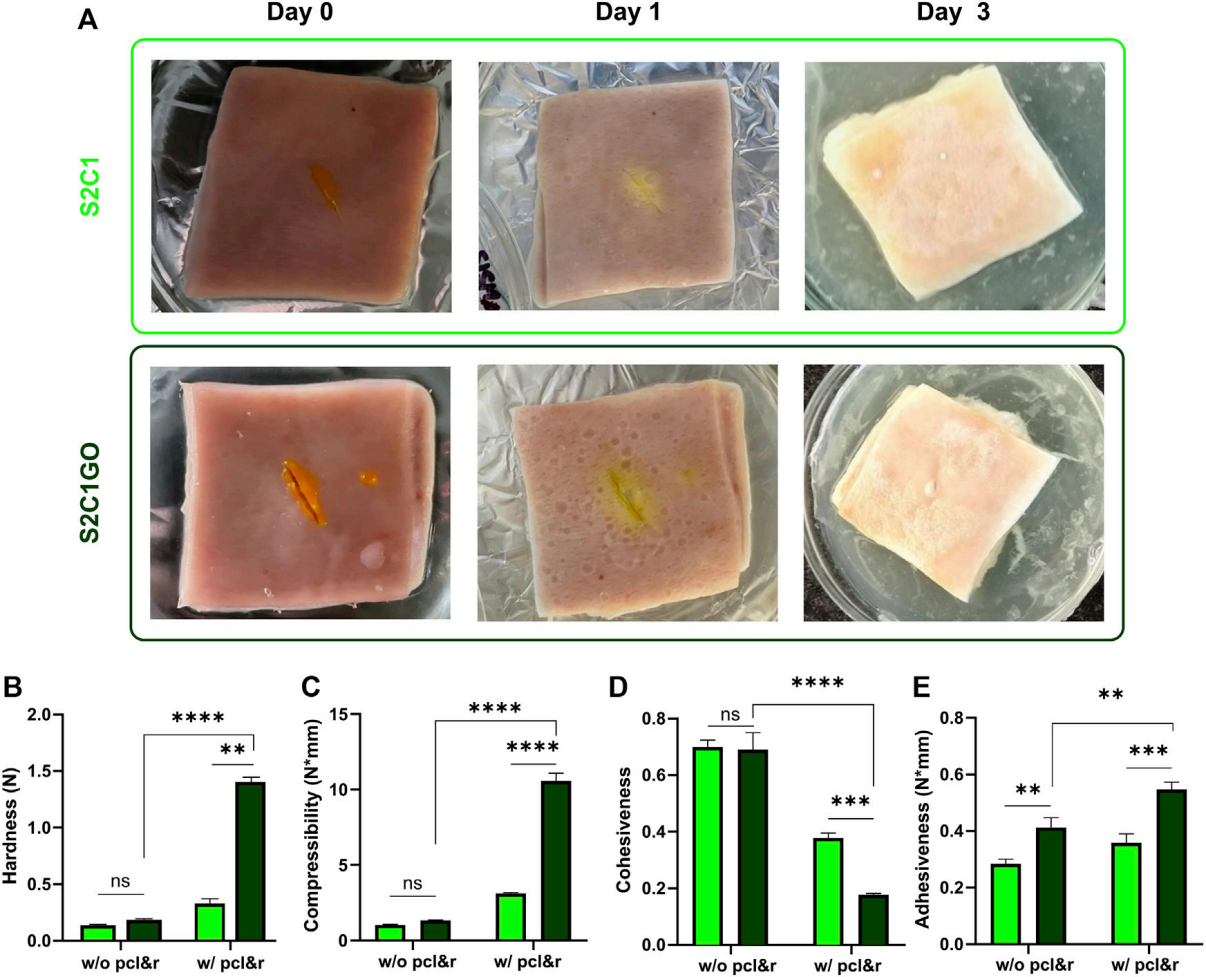
Mechanical and texture evaluation of the SISMA 2%/ChiMA 1% (S2C1) and SISMA 2%/ChiMA 1%/graphene oxide (S2C1GO) nanocomposite hydrogels. **(A)** Qualitative adhesion of skin cut wounds at 0, 1, and 3 days after application of the hydrogels. **(B)** Hardness, **(C)** compressibility, **(D)** cohesiveness, and **(E)** adhesiveness of the hydrogels before (w/o) and after (w/) photocrosslinking (pcl) and 2-weeks incubation (r) at 37°C.

The results of the qualitative and quantitative adhesion and texture analyses are presented in Figure 9A. Initially, adhesion was qualitatively assessed by sealing a cut wound opened on a fresh sample of porcine skin. Due to the inherent adhesiveness of methacrylate hydrogels, and specifically, the promising adhesiveness of ChiMA, both S2C1 and S2C1GO hydrogels provided adequate sealing for the wound. Not only did the hydrogels remain attached to the skin but were also capable of withstanding manual tearing loads. Qualitative adhesion was only monitored for 3 days, as the skin samples began to decompose under the employed incubation conditions.

Hardness, determined as the maximum peak force during the first compression cycle, was studied to measure the required force to produce the deformation of the hydrogels (Tuğcu-Demiröz, 2017). As evidenced in Figure 9B, S2C1GO samples yielded significantly higher values (*p* < 0.0001) than S2C1 samples, confirming GO’s contribution to the structural stability of the hydrogels after crosslinking. This shift from low to high hardness is a desirable behavior, as it indicates that unstimulated samples will be easily spread and applied to the skin. Also, the stimulated sample will have higher retention (Kakkar and Madhan, 2016).

Compressibility (Figure 9C) was estimated as the work required to deform the product during the first compression (Tuğcu-Demiröz, 2017). This property was significantly (*p* < 0.0001) enhanced by irradiation and reduction of the GO present in the S2C1GO samples, favoring the material’s potential to endure greater compression loads compared to S2C1 samples without and with crosslinking and incubation. As for the hardness, the shift from low to high values translates into ease of spreadability and superior resistance after irradiation (Tuğcu-Demiröz, 2017). The differences between the pristine hydrogel samples were non-statistically significant.

Cohesiveness reflects the reconstruction ability of gels after application and was determined as the ratio of the area under the force-time curve on the second compression cycle, and that produced on the first compression cycle (Figure 9D) (Tuğcu-Demiröz, 2017). The addition of GO failed to improve the material’s cohesiveness but maintain the ease of manipulation. Moreover, cohesiveness was significantly (*p* < 0.0001) reduced after crosslinking and reduction, limiting the structural recovery of the nanocomposite hydrogels (i.e., with dispersed rGO) (Karavana et al., 2009).

Adhesiveness represents the work required to overcome the attractive forces between the surfaces of the hydrogel and the test probe and was estimated as the negative force area for the first compression cycle (Figure 9E) (Tuğcu-Demiröz, 2017). The addition of GO significantly (*p* < 0.01) increases the hydrogel’s stickiness, thus improving its chances to attach over the skin surface (da Silva et al., 2018). This final improvement of adhesiveness suggests that the hydrogel’s application could potentially shorten the treatment period and consequently possibly improve the patient’s post-intervention recovery (Sezer et al., 2008).

### Hemolysis and Platelet Aggregation

**FIGURE 10:**
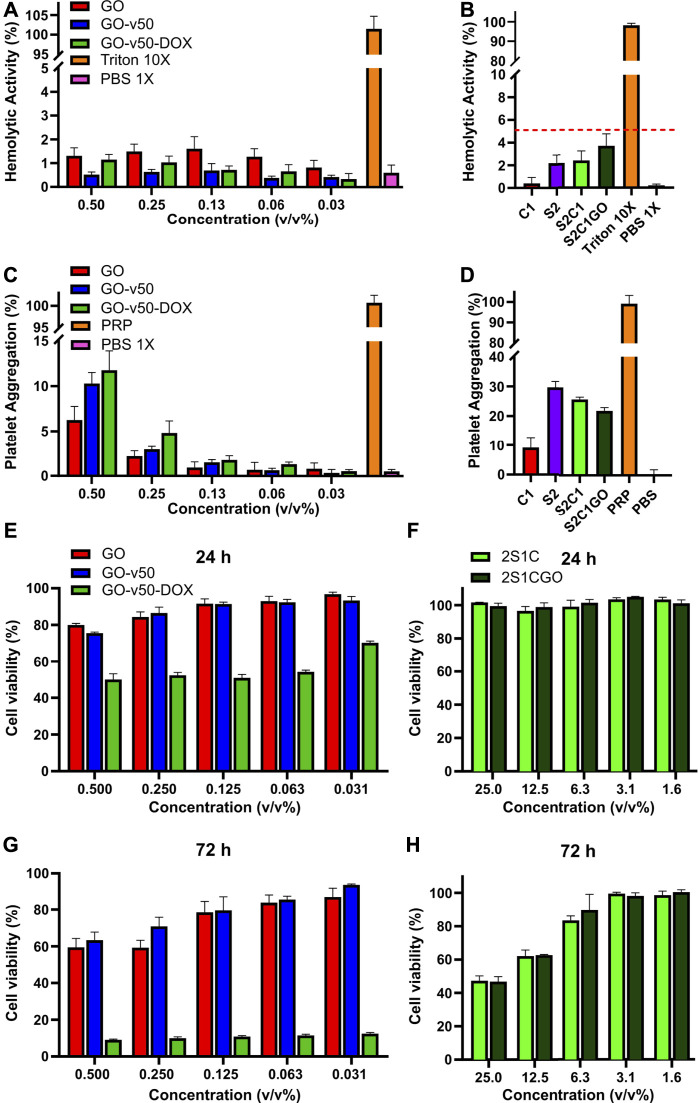
Biocompatibility evaluation of the pristine graphene oxide (GO), graphene oxide/v50 (GO-v50) and graphene oxide/v50/doxorubicin (GO-v50-DOX) nanoconjugates and the ChiMA 1% (C1), SISMA 2% (S2), SISMA 2%/ChiMA 1% (S2C1), and SISMA 2%/ChiMA 1%/graphene oxide (S2C1GO) hydrogels. Hemolytic activity of the **(A)** nanoconjugates and the **(B)** composite hydrogels, using triton and phosphate-buffered saline (PBS) as positive and negative controls. Platelet aggregation of the **(C)** nanoconjugates and the **(D)** composite hydrogels using platelet-rich plasma (PRP) and PBS as positive and negative controls. A-375 melanocytes’ viability evaluation after 24 h of exposure to the **(E)** GO-v50 and GO-v50-DOX nanoconjugates and the **(F)** nanocomposite hydrogels. Cell viability after 72 h of exposure to the **(G)** GO-v50 and GO-v50-DOX nanoconjugates and the **(H)** composite hydrogels.

Pristine and functionalized GO (i.e., GO, GO-v50, GO-v50-DOX) presented a non-hemolytic behavior, as evidenced by hemolytic activities below 2% for all the studied concentrations (Figure 10A). As free DOX has been reported to yield a stronger hemolytic tendency, these results suggest it remains inactive while conjugated to GO (Khan et al., 2015). Similarly, the evaluated hydrogels (S2C1 and S2C1GO) and their controls (S2 and C1) yielded hemolytic activities below 5% (Figure 10B). Although GO has been reported to exhibit dose-dependent toxicity toward erythrocytes, these results show that this undesirable feature was suppressed by both its addition in non-lethal doses and the high biocompatibility of the base hydrogel matrix (Vallabani et al., 2011; Figueroa et al., 2020).

Platelet aggregation was dose-dependent for pristine and functionalized GO, as aggregation decreased after each dilution. Although aggregation increased as functionalization proceeded, none of the functionalized samples surpassed 12%, and thus they can be classified as low aggregants (Figure 10C). Additionally, hydrogels based on SISMA yielded aggregation higher than 20%, implying they can be classified as medium aggregants (Figure 10D). Although higher aggregation was expected, recent reports have suggested that methacryloyl modifications may inhibit platelet aggregation by inactivating the polymeric backbones of SIS and chitosan (Chou et al., 2003; Blinc et al., 2004). Nevertheless, the achieved aggregation with S2C1GO is sufficient to create chemotactic gradients that favor the recruitment of stem cells, stimulate cell migration and differentiation, and promote tissue repair, thereby making the material a suitable wound dressing for post-treatment recovery (Nurden, 2008).

### Cytotoxicity

Results of MTT assays for pristine and functionalized GO revealed that even though GO and v50 have no important impact on cell viability, DOX proves to be a highly cytotoxic agent towards A-375 melanocytes. The exposure to GO-v50-DOX had a significant effect (*p* < 0.0001) in cell viability which dropped to about 50% after 24 h and to about 10% after 72 h, that can be most likely due to the passive release of DOX from the nanoconjugate as time passed (Figure 10E). Moreover, the cytotoxic effect of DOX remained unaffected despite dose reduction (*p* > 0.0001), while GO and v50 were hindered which each subsequent dilution. Similarly, S2C1 and S2C1GO hydrogels exhibited promising cytocompatibility (Figure 10F), as all the evaluated concentrations yielded none significant (*p* > 0.001) dose-dependent viability levels over 90% after 24 h and over 40% after 72 h. Importantly, microplate assays *in vitro* may broadly predict material-culture interactions, but fail to represent clearance phenomena, and thus, hydrogel components like riboflavin may accumulate in highly toxic doses towards a non-representative population of cells (Flieger et al., 2016). Nevertheless, the nanocomposite hydrogel succeeds in limiting the basal cytotoxic effect of the GO-v50-DOX nanoconjugate, thereby suggesting that cell death will only occur under the photo-induced release of DOX.

### *In silico* Analysis of the Chemo/Photothermal Therapy

**FIGURE 11:**
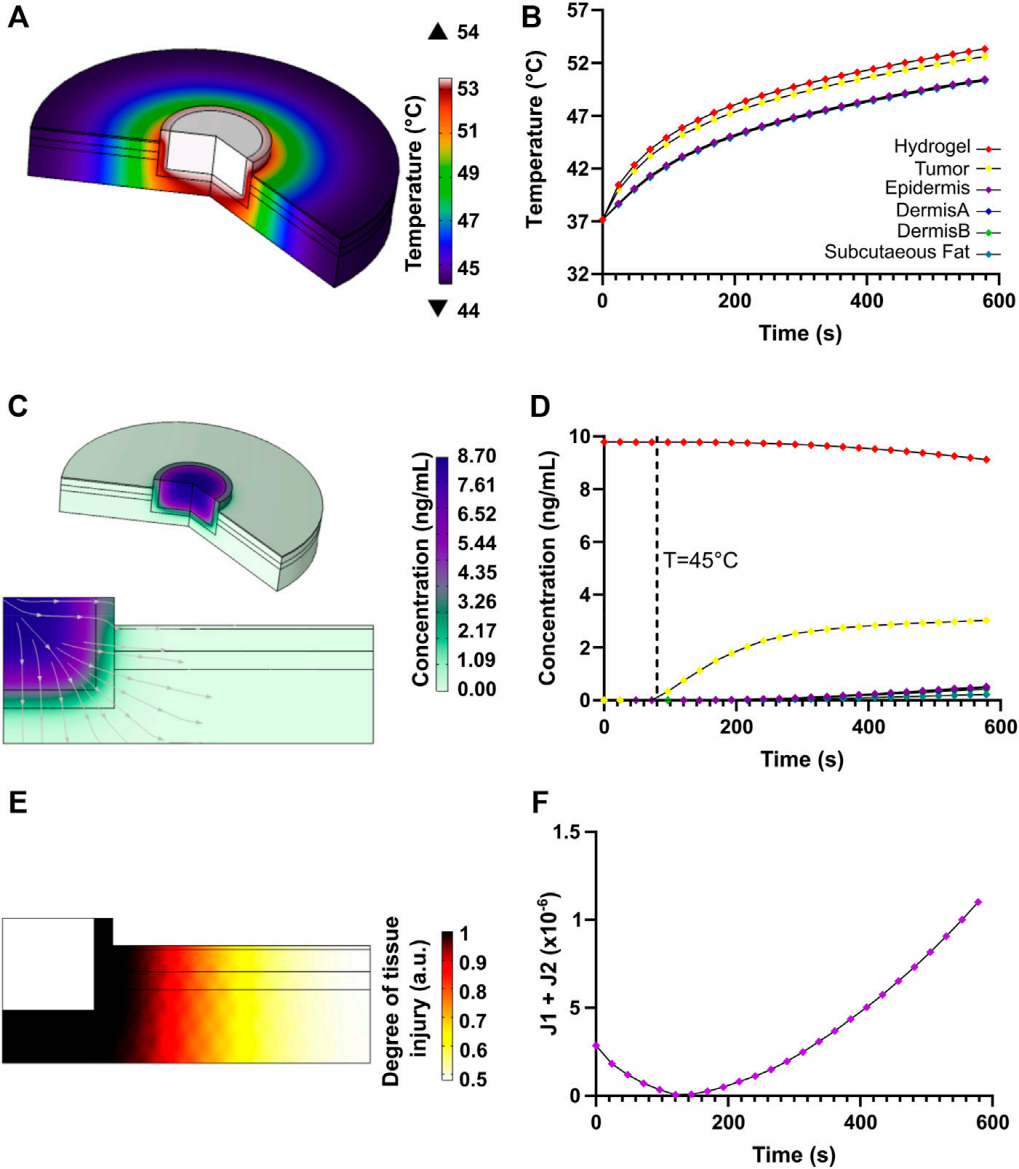
Heat transfer and transport of diluted species results for the chemo/photothermal therapy. **(A)** Temperature profile due to NIR-induced heating in the skin multilayer model. **(B)** Heat transport with respect to time at locations of interest along with the different components of the computational domain. **(C)** Doxorubicin release profile and streamlines. **(D)** Time evolution of doxorubicin mass diffusion after thermo-linker breakup. **(E)** Degree of tissue injury due to NIR-induced injury as calculated by the Henriques-Moritz equation. **(F)** Optimization function for heating the tumor tissue relative to the healthy tissue.

Heat transfer simulation results for the photothermal therapy are shown in Figure 11. The temperature profile shows that the heat is mainly localized where the nanocomposite hydrogel was deposited along with the tumor remnants, but gradually attenuates along with the surrounding healthy tissue (Figure 11A). Figure 11B shows the temporal evolution of temperature at the hydrogel nanocomposite, tumor tissue, and skin multilayer model domains. The temperature raised sharply during the first 200 s of photothermal therapy, then steadily increased and stabilized after 300 s. According to Figure 11B, a maximum temperature of 54°C is achieved at the center of the hydrogel computational domain. The residual tumor cells were exposed to higher temperatures (∼50°C) than the healthy tissue cells because they were close to the heated hydrogel nanocomposite. The temperature values reached within the tumor exceeded the threshold for protein denaturation and the activation of the cell-death pathway, which has been reported to be above 42.5°C (Wust et al., 2002; He et al., 2006; Simanovskii et al., 2006). In contrast, the surrounding healthy tissue reached temperatures ranging from 44 to 47°C, which is insufficient to produce tissue damage (Xu et al., 2019).

Figure 11E shows the degree of tissue injury caused by hyperthermia achieved with the proposed therapy. The critical value, Ω = 1, implies that enough permanent heat damage took place (Henriques and Moritz, 1947; Ani et al., 2018). The Arrhenius equation was originally related to the percentage of a volume of cells surviving a uniform temperature exposure for some time (Ani et al., 2018). It was evidenced that damage mainly focused on the malignant tissue and spread, to a lesser extent, towards healthy tissue close to the tumor, leading to insignificant damage. Our findings reveal that deleterious healthy tissue damage is highly limited if heating lasts for about 160 s, implying that the tumor tissue can be potentially eliminated with high efficiency.

Regarding the release of DOX after the thermo-linker breakdown, a mass diffusion profile corroborates that the drug concentration is higher within the tumor region, which is beneficial to complement thermal therapy and increase the eradication of cancerous tissue remnants (Figure 11C). Likewise, it is possible to observe that only a low concentration of the drug reached small portions of healthy tissue, thereby reducing the possible undesirable damage. Figure 11D shows that as a function of time the concentration of released DOX was higher in the tumor than in the skin tissue, which provides further evidence for the therapy’s high effectiveness. All in all, the combined effects of higher drug concentration and highly localized hyperthermia in tumor tissue provided by our therapy are anticipated to have a positive impact and improve clinical outcomes as an alternative to current approaches for melanoma treatment (Urano, 1999; Wust et al., 2002).

### Therapy

**FIGURE 12:**
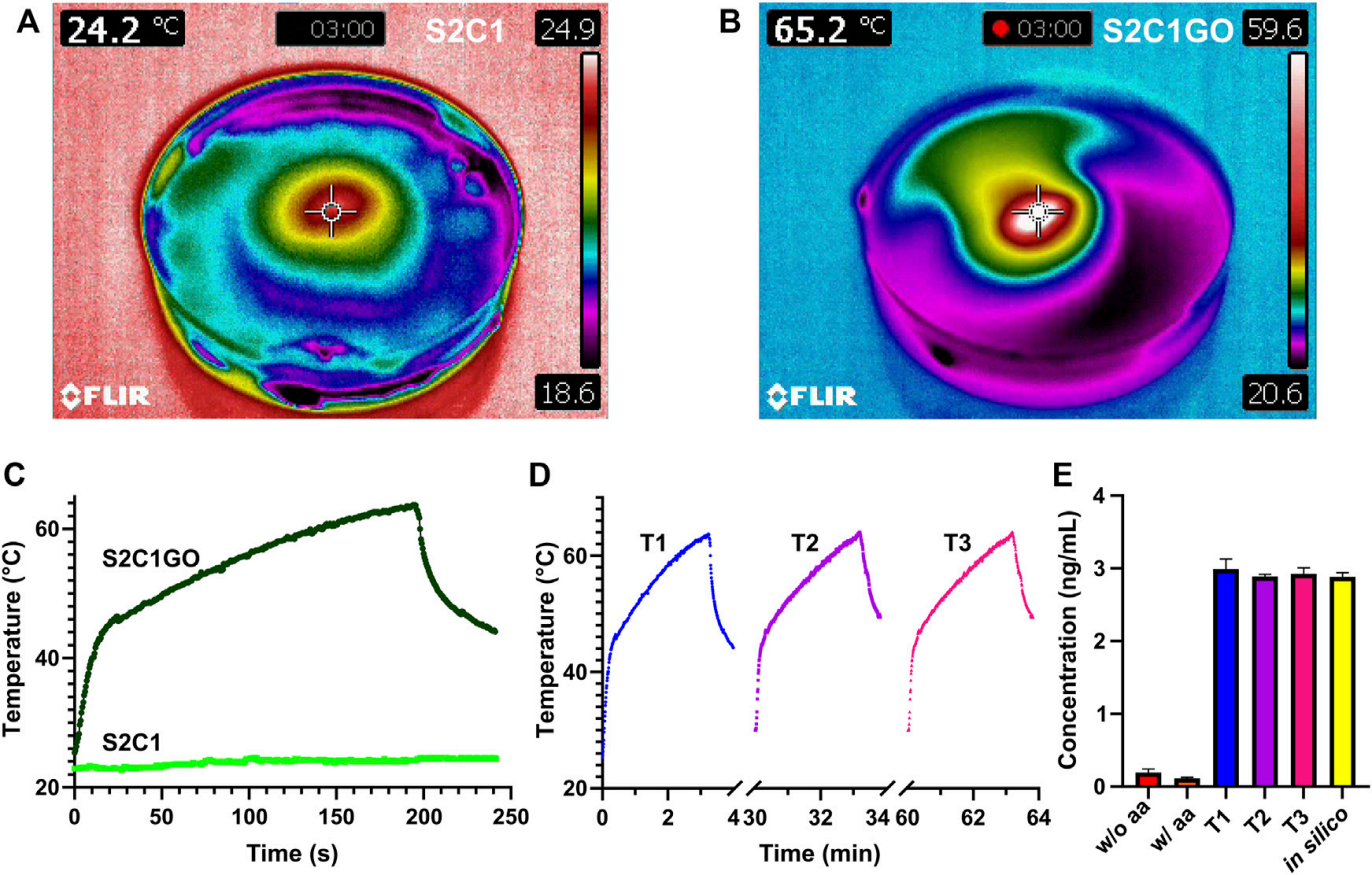
*In vitro* evaluation of the chemo/photothermal therapy. The temperature profile of **(A)** SISMA 2%/ChiMA 1% (S2C1) and **(B)** SISMA 2%/ChiMA 1%/graphene oxide (S2C1GO) hydrogels after 3 min irradiation with NIR. Photothermal heating of S2C1 and S2C1GO during **(C)** a first therapy cycle (T1) and **(D)** subsequent second (T2) and third (T3) therapy cycles. **(E)** Concentration of DOX released from S2C1GO for the different therapies using simulations (in silico) and unirradiated hydrogels with (w/) and without (w/o) ascorbic acid (aa) as positive and negative controls.

Results for the *in vitro* evaluation of the proposed chemo/photothermal therapy are presented in Figure 12. Temperature changes during irradiation confirm that rGO serves as the active PTA responsible for the hydrogels heating, as evidenced by the 62°C achieved with S2C1GO in under 3 min. In contrast, S2C1 barely increased 2°C with respect to the initial temperature in the same timeframe. Additionally, the temperature distribution (Figure 12B) evidenced that hyperthermia is restricted to the nanocomposite hydrogel vicinity since heat transfer is only favored by rGO’s thermal conductivity. No significant changes in the photothermal conversion of rGO were observed between therapies with different numbers of cycles (Figure 12D), indicating that the material’s photothermal capacity remains even after repeated stimulation. However, repeated stimulation was expected to promote even more heating as the cycles progress, since rGO might further reduce when exposed to heat, and thus, the lack of enhanced heating suggests that the material’s reduction in the presence of ascorbic acid was very efficient. Additionally, since photothermal heating surpasses the threshold of 45°C within the first minute, the v50 linker was successfully destabilized and DOX was released into the surrounding medium, achieving a concentration of about 3 ng/ml (Figure 12E). For the different treatments, however, no significant changes in the DOX concentration achieved were identified, and controls suggest that no DOX was passively released into the media during the first 8 h of incubation. The simulation results agree well with those obtained experimentally.

## Conclusion

Despite remarkable advancements in surgical techniques for melanoma tumor resection, cancer recurrence from the proliferation of undetectable microtumor residues remains a concern. In this work, we put forward a SISMA/ChiMA/rGO-DOX nanocomposite smart hydrogel capable of both generating local hyperthermia and releasing DOX under NIR light stimuli. By adding methacryloyl groups to the polymeric structure, we synthesized a material suitable for *in situ* deposition and crosslinking, and by dispersing ascorbic acid into the hydrated structure, we yield a material capable of self-reducing GO at body temperature. Moreover, we modified GO into a vehicle that temporarily binds and inactivates DOX and dispersed it into a biomimetic matrix with wound healing potential, thus achieving a multifunctional platform for stimuli-dependent drug delivery and post-treatment recovery. Furthermore, we put forward an *in silico* model in agreement with experimental results, which enabled us to plan treatments to maximize tumor death and minimize unspecific damage to healthy tissue. We are confident that these results pave the way for the development of new multifunctional materials for novel applications in cancer treatments. Future work should focus on its further validation *in vivo*.

## Data Availability

The original contributions presented in the study are included in the article/Supplementary Material, further inquiries can be directed to the corresponding authors.

## References

[B1] Ani C. J. Danyuo Y. Odusanya O. S. Soboyejo W. O. (2018). Computational Modeling of Drug Diffusion and Inductive Heating in an Implantable Biomedical Device for Localized Thermo-Chemotherapy of Cancer Cells/tissue. Cogent Eng. 5, 1463814. 10.1080/23311916.2018.1463814

[B2] Askari E. Seyfoori A. Amereh M. Gharaie S. S. Ghazali H. S. Ghazali Z. S. (2020). Stimuli-Responsive Hydrogels for Local Post-Surgical Drug Delivery. Gels 6, 14. 10.3390/gels6020014 PMC7345431 32397180

[B3] da Silva J. B. Ferreira S. Reis A. Cook M. Bruschi M. (2018). Assessing Mucoadhesion in Polymer Gels: The Effect of Method Type and Instrument Variables. Polymers 10, 254. 10.3390/polym10030254 PMC6415125 30966289

[B4] Blanpain C. Fuchs E. (2009). Epidermal Homeostasis: a Balancing Act of Stem Cells in the Skin. Nat. Rev. Mol. Cell Biol. 10, 207–217. 10.1038/nrm2636 19209183 PMC2760218

[B5] Blinc A. Božič M. Vengust R. Stegnar M. (2004). Methyl-methacrylate Bone Cement Surface Does Not Promote Platelet Aggregation or Plasma Coagulation *in Vitro* . Thrombosis Res. 114, 179–184. 10.1016/j.thromres.2004.05.010 15342214

[B6] Campbell E. Hasan M. T. Pho C. Callaghan K. Akkaraju G. R. Naumov A. V. (2019). Graphene Oxide as a Multifunctional Platform for Intracellular Delivery, Imaging, and Cancer Sensing. Sci. Rep. 9, 416. 10.1038/s41598-018-36617-4 30674914 PMC6344482

[B7] Çatıkkaş B. (2017). Raman and FT-IR Spectra, DFT and SQMFF Calculations for N,N-Dimethylaniline. Periodicals Eng. Nat. Sci. 5, 237–244. 10.21533/pen.v5i2.139

[B8] Céspedes-Valenzuela D. N. Sánchez-Rentería S. Cifuentes J. Gantiva-Diaz M. Serna J. A. Reyes L. H. (2021a). Preparation and Characterization of an Injectable and Photo-Responsive Chitosan Methacrylate/Graphene Oxide Hydrogel: Potential Applications in Bone Tissue Adhesion and Repair. Polymers 14, 126. 10.3390/polym14010126 35012148 PMC8747203

[B9] Céspedes-Valenzuela D. N. Sánchez-Rentería S. Munoz-Camargo C. Cruz J. C. (2021b). “Chitosan Methacrylate-Based Bioadhesive: towards a Photoresponsive and Extrudable Material for Bone Fracture Repair,” in 2021 IEEE 2nd International Congress of Biomedical Engineering and Bioengineering (CI-IB&BI), Bogota D.C., Colombia, 13-15 October (IEEE). 10.1109/ci-ibbi54220.2021.9626101

[B10] Chen Y. Yang Y. Xian Y. Singh P. Feng J. Cui S. (2019). Multifunctional Graphene-Oxide-Reinforced Dissolvable Polymeric Microneedles for Transdermal Drug Delivery. ACS Appl. Mat. Interfaces 12, 352–360. 10.1021/acsami.9b19518 31825580

[B11] Chou T.-C. Fu E. Wu C.-J. Yeh J.-H. (2003). Chitosan Enhances Platelet Adhesion and Aggregation. Biochem. Biophysical Res. Commun. 302, 480–483. 10.1016/s0006-291x(03)00173-6 12615058

[B12] Coit D. G. Thompson J. A. Albertini M. R. Barker C. Carson W. E. Contreras C. (2019). Cutaneous Melanoma, Version 2.2019, NCCN Clinical Practice Guidelines in Oncology. J. Natl. Compr. Cancer Netw. 17, 367–402. 10.6004/jnccn.2019.0018 30959471

[B13] Coussot P. (2017). “Introduction to Rheology and Fluid Mechanics,” in Mudflow Rheology and Dynamics (England: Routledge), 25–44. 10.1201/9780203746349-2

[B14] Cummins D. L. Cummins J. M. Pantle H. Silverman M. A. Leonard A. L. Chanmugam A. (2006). Cutaneous Malignant Melanoma. Mayo Clin. Proc. 81, 500–507. 10.4065/81.4.500 16610570

[B15] Dadsetan M. Liu Z. Pumberger M. Giraldo C. V. Ruesink T. Lu L. (2010). A Stimuli-Responsive Hydrogel for Doxorubicin Delivery. Biomaterials 31, 8051–8062. 10.1016/j.biomaterials.2010.06.054 20696470 PMC2936247

[B16] Dash B. S. Jose G. Lu Y.-J. Chen J.-P. (2021). Functionalized Reduced Graphene Oxide as a Versatile Tool for Cancer Therapy. Int. J. Mol. Sci. 22, 2989. 10.3390/ijms22062989 33804239 PMC8000837

[B17] DeNardo G. L. DeNardo S. J. (2008). Update: Turning the Heat on Cancer. Cancer Biotherapy Radiopharm. 23, 671–680. 10.1089/cbr.2008.0591 PMC2987268 20443694

[B18] Eda G. Chhowalla M. (2010). Chemically Derived Graphene Oxide: Towards Large-Area Thin-Film Electronics and Optoelectronics. Adv. Mat. 22, 2392–2415. 10.1002/adma.200903689 20432408

[B19] Farhane Z. Bonnier F. Casey A. Byrne H. J. (2015). Raman Micro Spectroscopy for *in Vitro* Drug Screening: Subcellular Localisation and Interactions of Doxorubicin. Analyst 140, 4212–4223. 10.1039/c5an00256g 25919793

[B20] Feng H. Cheng R. Zhao X. Duan X. Li J. (2013). A Low-Temperature Method to Produce Highly Reduced Graphene Oxide. Nat. Commun. 4, 1539. 10.1038/ncomms2555 23443567

[B21] Fernandez‐Villamarin M. Brooks L. Mendes P. M. (2019). The Role of Photochemical Reactions in the Development of Advanced Soft Materials for Biomedical Applications. Adv. Opt. Mat. 7, 1900215. 10.1002/adom.201900215

[B22] Figueroa T. Aguayo C. Fernandez K. (2020). Design and Characterization of Chitosan-Graphene Oxide Nanocomposites for the Delivery of Proanthocyanidins. Int. J. Nanomedicine 15, 1229–1238. 10.2147/ijn.s240305 32110019 PMC7039064

[B23] Flieger M. Bandouchova H. Cerny J. Chudíčková M. Kolarik M. Kovacova V. (2016). Vitamin B2 as a Virulence Factor in Pseudogymnoascus Destructans Skin Infection. Sci. Rep. 6, 33200. 10.1038/srep33200 27620349 PMC5020413

[B24] He Y. Shirazaki M. Liu H. Himeno R. Sun Z. (2006). A Numerical Coupling Model to Analyze the Blood Flow, Temperature, and Oxygen Transport in Human Breast Tumor under Laser Irradiation. Comput. Biol. Med. 36, 1336–1350. 10.1016/j.compbiomed.2005.08.004 16263105

[B25] Henriques F. C. Moritz A. R. (1947). Studies of Thermal Injury: I. The Conduction of Heat to and through Skin and the Temperatures Attained Therein. A Theoretical and an Experimental Investigation. Am. J. Pathol. 23, 530–549. 19970945 PMC1934298

[B26] Hocevar M. Dragonja Z. Pilko G. Gazic B. Zgajnar J. (2014). Residual Melanoma after an Excisional Biopsy Is an Independent Prognostic Factor for Local Recurrence and Overall Survival. Eur. J. Surg. Oncol. (EJSO) 40, 1271–1275. 10.1016/j.ejso.2014.03.002 24656456

[B27] Hou X. Pang Y. Li X. Yang C. Liu W. Jiang G. (2019). Core-shell T-ype T-hermo-nanoparticles L-oaded with T-emozolomide C-ombined with P-hotothermal T-herapy in M-elanoma C-ells. Oncol. Rep. 42, 2512. 10.3892/or.2019.7329 31545500 PMC6826326

[B28] Hu J.-J. Cheng Y.-J. Zhang X.-Z. (2018). Recent Advances in Nanomaterials for Enhanced Photothermal Therapy of Tumors. Nanoscale 10, 22657–22672. 10.1039/c8nr07627h 30500042

[B29] Huang S. Liu H. Huang S. Fu T. Xue W. Guo R. (2020). Dextran Methacrylate Hydrogel Microneedles Loaded with Doxorubicin and Trametinib for Continuous Transdermal Administration of Melanoma. Carbohydr. Polym. 246, 116650. 10.1016/j.carbpol.2020.116650 32747282

[B30] Jafarigol E. Salehi M. B. Mortaheb H. R. (2020). Preparation and Assessment of Electro-Conductive Poly(acrylamide-Co-Acrylic Acid) Carboxymethyl Cellulose/reduced Graphene Oxide Hydrogel with High Viscoelasticity. Chem. Eng. Res. Des. 162, 74–84. 10.1016/j.cherd.2020.07.020

[B31] Johnson-Arbor K. Duber R. (2021). Doxorubicin. Treasure Island: StatPearls Publishing. 29083582

[B32] Joyce D. Skitzki J. J. (2020). Surgical Management of Primary Cutaneous Melanoma. Surg. Clin. N. Am. 100, 61–70. 10.1016/j.suc.2019.09.001 31753116

[B33] Kakkar P. Madhan B. (2016). Fabrication of Keratin-Silica Hydrogel for Biomedical Applications. Mater. Sci. Eng. C 66, 178–184. 10.1016/j.msec.2016.04.067 27207052

[B34] Kankala R. K. Liu C.-G. Yang D.-Y. Wang S.-B. Chen A.-Z. (2020). Ultrasmall Platinum Nanoparticles Enable Deep Tumor Penetration and Synergistic Therapeutic Abilities through Free Radical Species-Assisted Catalysis to Combat Cancer Multidrug Resistance. Chem. Eng. J. 383, 123138. 10.1016/j.cej.2019.123138

[B35] Karki N. Tiwari H. Tewari C. Rana A. Pandey N. Basak S. (2020). Functionalized Graphene Oxide as a Vehicle for Targeted Drug Delivery and Bioimaging Applications. J. Mat. Chem. B 8, 8116–8148. 10.1039/d0tb01149e 32966535

[B36] Khan M. A. N. Aljarbou A. H. Aldebasi Y. S. Alorainy M. Khan A. (2015). Combination of Glycosphingosomes and Liposomal Doxorubicin Shows Increased Activity against Dimethyl-&alpha;-Benzanthracene-Induced Fibrosarcoma in Mice. Int. J. Nanomedicine 10, 6331. 10.2147/ijn.s86467 26504383 PMC4605236

[B37] Kim E. Kim M. H. Song J. H. Kang C. Park W. H. (2020). Dual Crosslinked Alginate Hydrogels by Riboflavin as Photoinitiator. Int. J. Biol. Macromol. 154, 989–998. 10.1016/j.ijbiomac.2020.03.134 32194119

[B38] Kim H. Lee D. Kim J. Kim T.-i. Kim W. J. (2013). Photothermally Triggered Cytosolic Drug Delivery via Endosome Disruption Using a Functionalized Reduced Graphene Oxide. ACS Nano 7, 6735–6746. 10.1021/nn403096s 23829596

[B39] Krishnamoorthy K. Veerapandian M. Mohan R. Kim S.-J. (2011). Investigation of Raman and Photoluminescence Studies of Reduced Graphene Oxide Sheets. Appl. Phys. A 106, 501–506. 10.1007/s00339-011-6720-6

[B40] Krishnamoorthy K. Veerapandian M. Yun K. Kim S.-J. (2013). The Chemical and Structural Analysis of Graphene Oxide with Different Degrees of Oxidation. Carbon 53, 38–49. 10.1016/j.carbon.2012.10.013

[B41] Krylova V. Dukštienė N. (2019). The Structure of PA-Se-S-Cd Composite Materials Probed with FTIR Spectroscopy. Appl. Surf. Sci. 470, 462–471. 10.1016/j.apsusc.2018.11.121

[B42] Li J. Mooney D. J. (2016). Designing Hydrogels for Controlled Drug Delivery. Nat. Rev. Mater 1, 16071. 10.1038/natrevmats.2016.71 29657852 PMC5898614

[B43] Lima-Sousa R. de Melo-Diogo D. Alves C. G. Cabral C. S. D. Miguel S. P. Mendonça A. G. (2020). Injectable *In Situ* Forming Thermo-Responsive Graphene Based Hydrogels for Cancer Chemo-Photothermal Therapy and NIR Light-Enhanced Antibacterial Applications. Mater. Sci. Eng. C 117, 111294. 10.1016/j.msec.2020.111294 32919655

[B44] Lopez-Barbosa N. Suárez-Arnedo A. Cifuentes J. Gonzalez Barrios A. F. Silvera Batista C. A. Osma J. F. (2019). Magnetite-OmpA Nanobioconjugates as Cell-Penetrating Vehicles with Endosomal Escape Abilities. ACS Biomater. Sci. Eng. 6, 415–424. 10.1021/acsbiomaterials.9b01214 33463215

[B45] Marcano D. C. Kosynkin D. V. Berlin J. M. Sinitskii A. Sun Z. Slesarev A. (2010). Improved Synthesis of Graphene Oxide. ACS Nano 4, 4806–4814. 10.1021/nn1006368 20731455

[B46] Muzyka R. Drewniak S. Pustelny T. Chrubasik M. Gryglewicz G. (2018). Characterization of Graphite Oxide and Reduced Graphene Oxide Obtained from Different Graphite Precursors and Oxidized by Different Methods Using Raman Spectroscopy. Materials 11, 1050. 10.3390/ma11071050 PMC6073803 29933564

[B47] Nosrati A. Berliner J. G. Goel S. McGuire J. Morhenn V. de Souza J. R. (2017). Outcomes of Melanoma *In Situ* Treated with Mohs Micrographic Surgery Compared with Wide Local Excision. JAMA Dermatol 153, 436. 10.1001/jamadermatol.2016.6138 28241261 PMC5817486

[B48] Nurden A. T. (2008). Platelets and Wound Healing. Front. Biosci. 13, 3532. 10.2741/2947 18508453

[B49] Patarroyo J. L. Fonseca E. Cifuentes J. Salcedo F. Cruz J. C. Reyes L. H. (2021). Gelatin-Graphene Oxide Nanocomposite Hydrogels for Kluyveromyces Lactis Encapsulation: Potential Applications in Probiotics and Bioreactor Packings. Biomolecules 11, 922. 10.3390/biom11070922 34206397 PMC8302002

[B50] Paxton N. Smolan W. Böck T. Melchels F. Groll J. Jungst T. (2017). Proposal to Assess Printability of Bioinks for Extrusion-Based Bioprinting and Evaluation of Rheological Properties Governing Bioprintability. Biofabrication 9, 044107. 10.1088/1758-5090/aa8dd8 28930091

[B51] Pennes H. H. (1948). Analysis of Tissue and Arterial Blood Temperatures in the Resting Human Forearm. J. Appl. Physiology 1, 93–122. 10.1152/jappl.1948.1.2.93 18887578

[B52] Prickett K. A. Ramsey M. L. (2022). Mohs Micrographic Surgery. Treasure Island: StatPearls Publishing. 28722863

[B53] Raslan A. Saenz del Burgo L. Ciriza J. Pedraz J. L. (2020). Graphene Oxide and Reduced Graphene Oxide-Based Scaffolds in Regenerative Medicine. Int. J. Pharm. 580, 119226. 10.1016/j.ijpharm.2020.119226 32179151

[B54] Rebecca V. W. Somasundaram R. Herlyn M. (2020). Pre-clinical Modeling of Cutaneous Melanoma. Nat. Commun. 11, 2858. 10.1038/s41467-020-15546-9 32504051 PMC7275051

[B55] Rueda-Gensini L. Serna J. A. Cifuentes J. Cruz J. C. Muñoz-Camargo C. (2021). Graphene Oxide-Embedded Extracellular Matrix- Derived Hydrogel as a Multiresponsive Platform for 3D Bioprinting Applications. Int. J. Bioprint 7, 353. 10.18063/ijb.v7i3.353 34286147 PMC8287511

[B56] Sahraei R. Ghaemy M. (2017). Synthesis of Modified Gum Tragacanth/graphene Oxide Composite Hydrogel for Heavy Metal Ions Removal and Preparation of Silver Nanocomposite for Antibacterial Activity. Carbohydr. Polym. 157, 823–833. 10.1016/j.carbpol.2016.10.059 27987996

[B57] Sánchez-Palencia D. M. D׳Amore A. González-Mancera A. Wagner W. R. Briceño J. C. (2014). Effects of Fabrication on the Mechanics, Microstructure and Micromechanical Environment of Small Intestinal Submucosa Scaffolds for Vascular Tissue Engineering. J. Biomechanics 47, 2766–2773. 10.1016/j.jbiomech.2014.04.048 24877881

[B58] Sandolo C. Matricardi P. Alhaique F. Coviello T. (2009). Effect of Temperature and Cross-Linking Density on Rheology of Chemical Cross-Linked Guar Gum at the Gel Point. Food Hydrocoll. 23, 210–220. 10.1016/j.foodhyd.2008.01.001

[B59] Sandru F. Draghici C. C. Predescu T. Constantin M. Petca R. C. Constantin T. (2020). Regressive Melanoma in a Female Patient: A Case Report. Exp. Ther. Med. 20, 87. 10.3892/etm.2020.8675 PMC7271722 32508999

[B60] Sarkar D. Haji-Sheikh A. Jain A. (2015). Temperature Distribution in Multi-Layer Skin Tissue in Presence of a Tumor. Int. J. Heat Mass Transf. 91, 602–610. 10.1016/j.ijheatmasstransfer.2015.07.089

[B61] Serna J. Florez S. Talero V. Briceño J. Muñoz-Camargo C. Cruz J. (2019). Formulation and Characterization of a SIS-Based Photocrosslinkable Bioink. Polymers 11, 569. 10.3390/polym11030569 PMC6473614 30960553

[B62] Sezer A. D. Cevher E. Hatıpoğlu F. Oğurtan Z. Baş A. L. Akbuğa J. (2008). Preparation of Fucoidan-Chitosan Hydrogel and its Application as Burn Healing Accelerator on Rabbits. Biol. Pharm. Bull. 31, 2326–2333. 10.1248/bpb.31.2326 19043221

[B63] Shao J. Ruan C. Xie H. Li Z. Wang H. Chu P. K. (2018). Black-Phosphorus-Incorporated Hydrogel as a Sprayable and Biodegradable Photothermal Platform for Postsurgical Treatment of Cancer. Adv. Sci. (Weinh) 5, 1700848. 10.1002/advs.201700848 29876210 PMC5978961

[B64] Sharma K. Kaith B. S. Kumar V. Kumar V. Kalia S. Kapur B. K. (2014). Corrigendum to: "A Comparative Study of the Effect of Ni9+ and Au8+ Ion Beams on the Properties of Poly(methacrylic Acid) Grafted Gum Ghatti Films [Radiat. Phys. Chem. 97 (2014) 253-261]". Radiat. Phys. Chem. 99, 97. 10.1016/j.radphyschem.2014.02.022

[B65] Shen Y. Tang H. Huang X. Hang R. Zhang X. Wang Y. (2020). DLP Printing Photocurable Chitosan to Build Bio-Constructs for Tissue Engineering. Carbohydr. Polym. 235, 115970. 10.1016/j.carbpol.2020.115970 32122504

[B66] Shenenberger D. W. (2012). Cutaneous Malignant Melanoma: A Primary Care Perspective. Am. Fam. Physician 85, 161–168. 22335216

[B67] Simanovskii D. M. Mackanos M. A. Irani A. R. O’Connell-Rodwell C. E. Contag C. H. Schwettman H. A. (2006). Cellular Tolerance to Pulsed Hyperthermia. Phys. Rev. E 74, 011915. 10.1103/physreve.74.011915 16907135

[B68] Song M. Yu L. Wu Y. (2012). Simple Synthesis and Enhanced Performance of Graphene Oxide-Gold Composites. J. Nanomater. 2012, 1–5. 10.1155/2012/135138

[B69] Stankovich S. Dikin D. A. Piner R. D. Kohlhaas K. A. Kleinhammes A. Jia Y. (2007). Synthesis of Graphene-Based Nanosheets via Chemical Reduction of Exfoliated Graphite Oxide. Carbon 45, 1558–1565. 10.1016/j.carbon.2007.02.034

[B70] Stobinski L. Lesiak B. Malolepszy A. Mazurkiewicz M. Mierzwa B. Zemek J. (2014). Graphene Oxide and Reduced Graphene Oxide Studied by the XRD, TEM and Electron Spectroscopy Methods. J. Electron Spectrosc. Relat. Phenom. 195, 145–154. 10.1016/j.elspec.2014.07.003

[B71] Strankowski M. Włodarczyk D. Piszczyk Ł. Strankowska J. (2016). Polyurethane Nanocomposites Containing Reduced Graphene Oxide, FTIR, Raman, and XRD Studies. J. Spectrosc. 2016, 1–6. 10.1155/2016/7520741

[B72] Taymaz-Nikerel H. Karabekmez M. E. Eraslan S. Kırdar B. (2018). Doxorubicin Induces an Extensive Transcriptional and Metabolic Rewiring in Yeast Cells. Sci. Rep. 8, 13672. 10.1038/s41598-018-31939-9 30209405 PMC6135803

[B73] Thomas D. J. King A. R. Peat B. G. (2003). Excision Margins for Nonmelanotic Skin Cancer. Plastic Reconstr. Surg. 112, 57–63. 10.1097/01.prs.0000067479.77859.31 12832877

[B74] Tohamy H.-A. S. El-Sakhawy M. Kamel S. (2020). Carboxymethyl Cellulose-Grafted Graphene Oxide/Polyethylene Glycol for Efficient Ni(II) Adsorption. J. Polym. Environ. 29, 859–870. 10.1007/s10924-020-01920-7

[B75] Tuğcu-demi̇röz F. (2017). Development of iitu Poloxamer-Chitosan Hydrogels for Vaginal Drug Delivery of Benzydamine Hydrochloride: Textural, Mucoadhesive and *in Vitro* Release Properties. Marmara Pharm. J. 21, 762–770. 10.12991/mpj.2017.3

[B76] Urano M. (1999). Invited Review: For the Clinical Application of Thermochemotherapy Given at Mild Temperatures. Int. J. Hyperth. 15, 79–107. 10.1080/026567399285765 10323618

[B77] Vallabani N. V. S. Mittal S. Shukla R. Pandey A. Dhakate S. Pasricha R. (2011). Toxicity of Graphene in Normal Human Lung Cells (BEAS-2B). J. Biomed. Nanotechnol. 7, 106–107. 10.1166/jbn.2011.1224 21485826

[B78] Vines J. B. Yoon J.-H. Ryu N.-E. Lim D.-J. Park H. (2019). Gold Nanoparticles for Photothermal Cancer Therapy. Front. Chem. 7, 167. 10.3389/fchem.2019.00167 31024882 PMC6460051

[B79] Wust P. Hildebrandt B. Sreenivasa G. Rau B. Gellermann J. Riess H. (2002). Hyperthermia in Combined Treatment of Cancer. Lancet Oncol. 3, 487–497. 10.1016/s1470-2045(02)00818-5 12147435

[B80] Xu Y. Long S. Yang Y. Zhou F. Dong N. Yan K. (2019). Mathematical Simulation of Temperature Distribution in Tumor Tissue and Surrounding Healthy Tissue Treated by Laser Combined with Indocyanine Green. Theor. Biol. Med. Model 16, 12. 10.1186/s12976-019-0107-3 31422770 PMC6699130

[B81] Yang H. Cai W. Lv W. Zhao P. Shen Y. Zhang L. (2019). A New Strategy for Accurate Targeted Diagnosis and Treatment of Cutaneous Malignant Melanoma: Dual-Mode Phase-Change Lipid Nanodroplets as Ultrasound Contrast Agents. Int. J. Nanomedicine 14, 7079–7093. 10.2147/ijn.s207419 31564866 PMC6731466

[B82] Yaprak Karavana S. Güneri P. Ertan G. (2009). Benzydamine Hydrochloride Buccal Bioadhesive Gels Designed for Oral Ulcers: Preparation, Rheological, Textural, Mucoadhesive and Release Properties. Pharm. Dev. Technol. 14, 623–631. 10.3109/10837450902882351 19883251

[B83] Yoo H. J. Mahapatra S. S. Cho J. W. (2014). High-Speed Actuation and Mechanical Properties of Graphene-Incorporated Shape Memory Polyurethane Nanofibers. J. Phys. Chem. C 118, 10408–10415. 10.1021/jp500709m

[B84] Zhang N. Song J. Liu Y. Liu M. Zhang L. Sheng D. (2019). Photothermal Therapy Mediated by Phase-Transformation Nanoparticles Facilitates Delivery of Anti-PD1 Antibody and Synergizes with Antitumor Immunotherapy for Melanoma. J. Control. Release 306, 15–28. 10.1016/j.jconrel.2019.05.036 31132380

[B85] Zhang X. Xu L. Wei S. Zhai M. Li J. (2012). Stimuli Responsive Deswelling of Radiation Synthesized Collagen Hydrogel in Simulated Physiological Environment. J. Biomed. Mat. Res. 101A, 2191–2201. 10.1002/jbm.a.34525 23281146

[B86] Zhu Q. Zhang A. Liu P. Xu L. X. (2012). Study of Tumor Growth under Hyperthermia Condition. Comput. Math. Methods Med. 2012, 1–9. 10.1155/2012/198145 PMC3438796 22973411

