# Supplementary material for: Novel Photo- and Thermo-Responsive Nanocomposite Hydrogels Based on Functionalized rGO and Modified SIS/Chitosan Polymers for Localized Treatment of Malignant Cutaneous Melanoma
Source: Front Bioeng Biotechnol. 2022 Jul 6;10:947616. doi: 10.3389/fbioe.2022.947616 (PMC9300866; doi:10.3389/fbioe.2022.947616)
Supplement: Supplementary file 1 [file DataSheet1.docx]

Supplementary Material


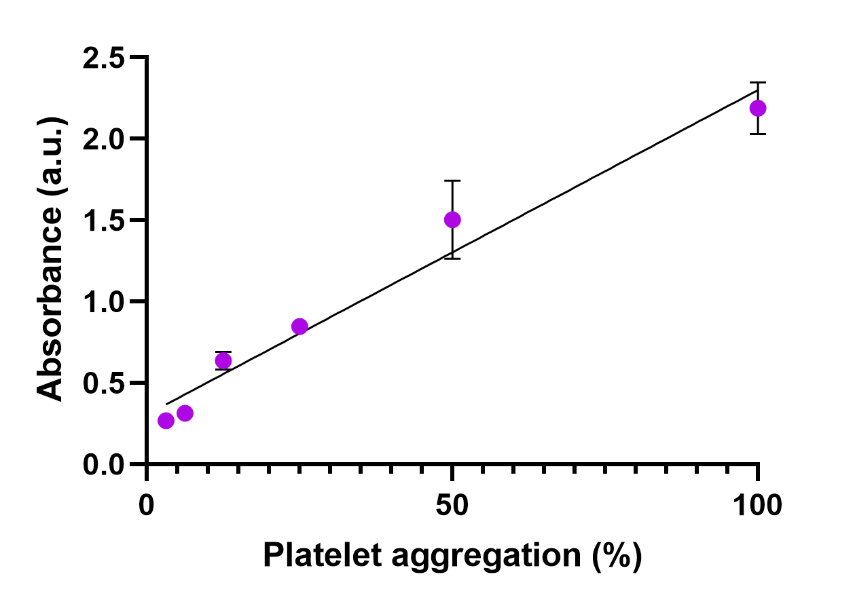


Supplementary Figure 1. Platelet aggregation calibration curve.

Supplementary Figure 2. FTIR spectra of the polymeric hydrogels.


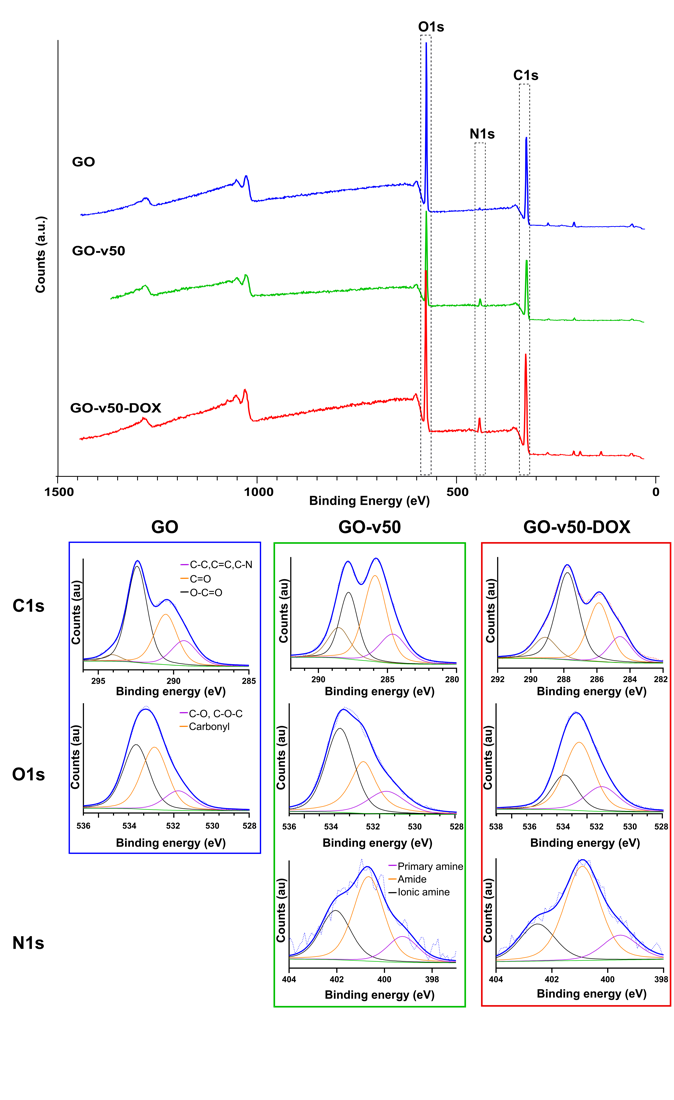


**Supplementary Figure 3.** XPS spectra of pristine and modified GO samples to assess the degree of substitution. (A) XPS narrow scan spectra of GO, GO-v50 and GO-v50-DOX samples for C1s, O1s and N1s signals. (B) The sub-peak components are shown under the fitted curves (blue line).


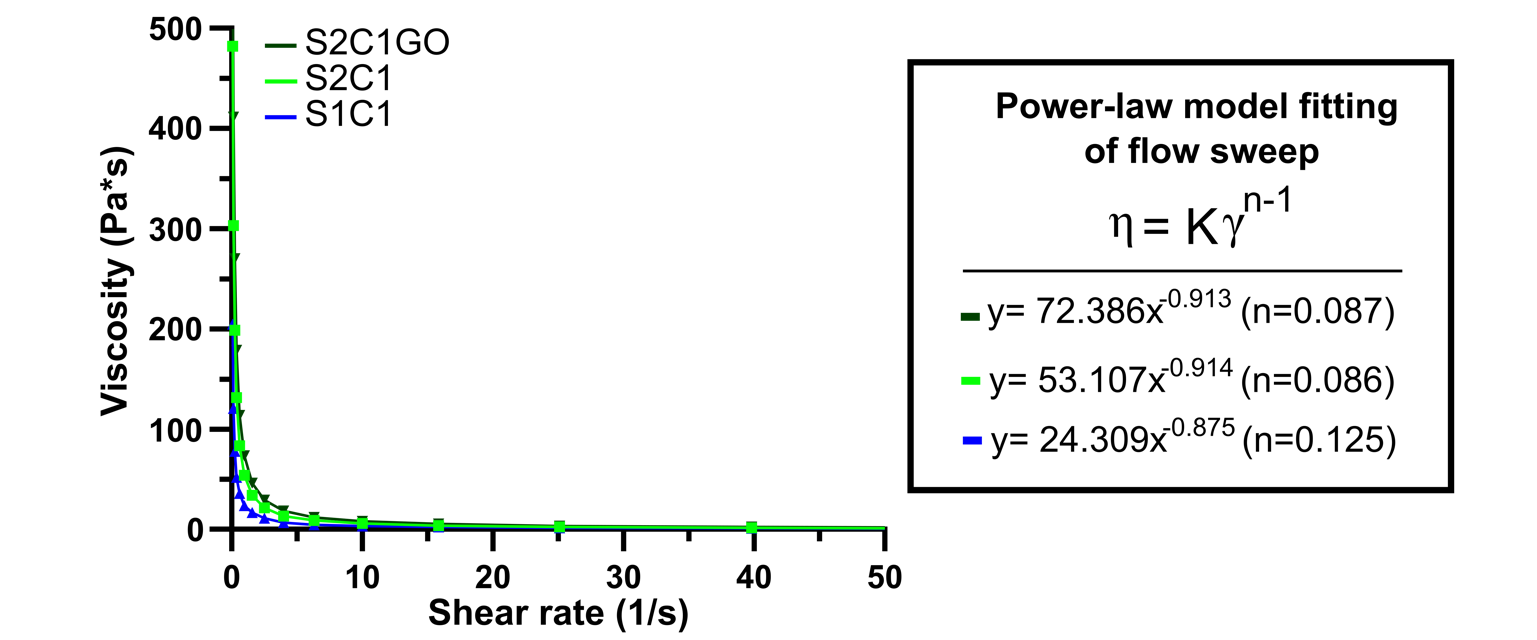


**Supplementary Figure 4.** Flow sweep power-law model fitting.
